# A MALDI-TOF MS database for fast identification of *Vibrio* spp. potentially pathogenic to marine mollusks

**DOI:** 10.1007/s00253-021-11141-0

**Published:** 2021-02-15

**Authors:** M. Moussa, E. Cauvin, A. Le Piouffle, O. Lucas, A. Bidault, C. Paillard, F. Benoit, B. Thuillier, M. Treilles, M. A. Travers, Céline Garcia

**Affiliations:** 1 Ifremer, SG2M-LGPMM, Laboratoire de Génétique et Pathologie des Mollusques Marins, F-17390 La Tremblade, France; 2 Labeo-Manche, 1352 avenue de Paris, 50000 Saint-Lô, France; 3 Labocea, Avenue de la Plage des Gueux, 29330 Quimper, France; 4 Qualyse, ZI Montplaisir, 79220 Champdeniers Saint-Denis, France; 5 Univ Brest, CNRS, IRD, Ifremer, UMR6539 LEMAR, F-29280 Plouzané, France; 6 IHPE, Univ. Montpellier, CNRS, Ifremer, Univ. Perpignan Via Domitia, F-34090 Montpellier, France

**Keywords:** *Vibrio*, MALDI-TOF MS, Database, Marine mollusk pathogens

## Abstract

**Abstract:**

In mollusk aquaculture, a large number of *Vibrio* species are considered major pathogens. Conventional methods based on DNA amplification and sequencing used to accurately identify *Vibrio* species are unsuitable for monitoring programs because they are time-consuming and expensive. The aim of this study was, therefore, to develop the MALDI-TOF MS method in order to establish a rapid identification technique for a large panel of *Vibrio* species. We created the EnviBase containing 120 main spectra projections (MSP) of the *Vibrio* species that are potentially responsible for mollusk diseases, comprising 25 species: *V. aestuarianus*, *V. cortegadensis*, *V. tapetis* and species belonging to the *Coralliilyticus*, *Harveyi*, *Mediterranei*, and *Orientalis* clades. Each MSP was constructed by the merger of raw spectra obtained from three different media and generated by three collaborating laboratories to increase the diversity of the conditions and thus obtain a good technique robustness. Perfect discrimination was obtained with all of the MSP created for the *Vibrio* species and even for very closely related species as *V. europaeus* and *V. bivalvicida*. The new EnviBase library was validated through a blind test on 100 *Vibrio* strains performed by our three collaborators who used the direct transfer and protein extraction methods. The majority of the *Vibrio* strains were successfully identified with the newly created EnviBase by the three laboratories for both protocol methods. This study documents the first development of a freely accessible database exclusively devoted to *Vibrio* found in marine environments, taking into account the high diversity of this genus.

**Key points:**

• *Development of a MALDI-TOF MS database to quickly affiliate Vibrio species.*

• *Increase of the reactivity when faced with Vibrio associated with mollusk diseases*.

• *Validation of MALDI-TOF MS as routine diagnostic tool*.

**Supplementary Information:**

The online version contains supplementary material available at 10.1007/s00253-021-11141-0.

## Introduction

The greatest worldwide threat to the future of marine aquaculture is disease. In particular, there are an increasing number of reports of bacterial infections impacting marine aquaculture production, as well as novel descriptions of new bacterial pathogens. A large number of bacterial species associated with marine organisms in symbiotic or pathogenic relationships belong to the genus *Vibrio* (Paillard et al. 2004; Thompson et al. 2004), which contains more than 100 species distributed across at least twenty clades (Al-Saari et al. 2015; Sawabe et al. 2013). Many *Vibrio* species are pathogenic for marine mollusks (Austin 2010; Paillard et al. 2004; Saulnier et al. 2010; Thompson et al. 2004; Travers et al. 2015), and some of these species can affect all of the growth stages (i.e., larval, juvenile, and adult) of their mollusk hosts, whereas others are more specialized (Beaz-Hidalgo et al. 2010; Paillard et al. 2004). Currently, these bacteria are recognized as the most diverse known marine bacterial group due to their presence in various niches in the environment, as well as their metabolic and ecologic versatility. Consequently, the characterization, classification, and identification of *Vibrio* species are particularly problematic, especially in very closely related species.

The development of molecular techniques such as multilocus sequence analysis (MLSA) (Thompson et al. 2005) has enabled the description of more than 50 species of *Vibrio*, helping to develop the phylogeny of the *Vibrionaceae* family. Given that 16S rRNA genes have revealed very few divergences between numerous *Vibrio* species (Le Roux et al. 2004; Sawabe et al. 2007), the identification of *Vibrio* is now mainly based on certain pertinent gene markers such as hsp60 (Hunt et al. 2008), multilocus sequencing (Sawabe et al. 2007, 2013), or complete genome sequencing (Lin et al. 2010; Tanaka et al. 2020).

The quantitative real-time PCR technique is already used for *Vibrio* identification (McCleary and Henshilwood 2015; Saulnier et al. 2009, 2017; Bidault et al. 2015). However, specific developments for each targeted pathogen are needed which could be both lengthy and costly. Moreover, qPCR recognizes and quantifies DNA of the target species, but does not imply viability or infection. Disease surveillance requires the use of fast and accurate diagnostic tools to identify (a) causative agent(s) in diseased individuals. Diagnostic tools are critical for early detection, especially in the absence of known clinical signs for marine mollusk diseases and because the causative agent could be exotic, emerging, or multifactorial (de Lorgeril et al. 2018; Lemire et al. 2015). Thus, wide-ranging approaches based on non-targeted tools are encouraged (Burge et al. 2016).

To improve the diagnostic technique and be able to be more reactive in the face of progressing diseases caused by *Vibrio* infections in mollusk aquaculture, it is important to explore new techniques that can be used to quickly screen the largest number of *Vibrio* species possible. For several years, MALDI-TOF MS (matrix-assisted laser desorption/ionization time-of-flight mass spectrometry) has emerged as a particularly powerful tool that is starting to be used in the field of routine microbiology in the agricultural chain. This proteomic method has proven to be reliable and safe for the identification of bacteria, yeasts, filamentous fungi, and dermatophytes (Carbonnelle et al. 2011, 2012; Gregory et al. 2018; Piamsomboon et al. 2020; Sloan et al. 2017; Tracz et al. 2017; Wang et al. 2002). MALDI-TOF-MS is a rapid, precise, and cost-effective identification method compared to conventional phenotypic techniques or molecular biology. Its ability to analyze whole microorganisms with little sample preparation has greatly reduced the time to identification (30 min). Compared with *Vibrio* species found in marine environments, pathogenic *Vibrio* species found in humans are widely represented in Bruker Daltonics library and other free MALDI-TOF databases (Erler et al. 2015, Ruvira et al. 2013). The aim of this study was to develop a MALDI-TOF *Vibrio* database, called the EnviBase, built on environmental, type strains and clinical strains of mollusk pathogens. By combining different culture conditions and sample preparation protocols, we generated 120 specific main spectra projections (MSP) for the *Vibrio* genus corresponding to 25 different species listed in a free database; we also validated this newly created database through a blind test. This work is the first study on the development of a database specifically intended to describe a high diversity of *Vibrio* species affecting marine organisms and which will enable an accurate rapid identification of a large number of *Vibrio* spp. in a very short time.

## Materials and methods

### *Vibrio* strains

A total of 220 strains of *Vibrio* spp. were analyzed (Table S1A-B, Table S3). Some of them originated from surveillance programs, primarily carried out in France from 2003 to 2016, in which they were isolated from a broad range of marine organisms such as oysters, cockles, and mussels and from the environment, in particular from seawater and sediments. Moreover, the type strain for each species was included in this study, except for *V. jasicida*. The strains were previously well characterized by PCR, through some housekeeping genes (*hsp60*, *gyrB*, *topA*, *pyrH*, *ldh*, *mreB*, *gapA*, *ftz*, *dnaJ*) and 16S rDNA gene sequencing, depending on the strains. The identification of certain species such as *V. aestuarianus*, *V. tapetis*, and *V. europaeus* was also confirmed by qPCR, as previously described (Bidault et al. 2015; Saulnier et al. 2009; Travers et al. 2014).

**Fig. 1:**
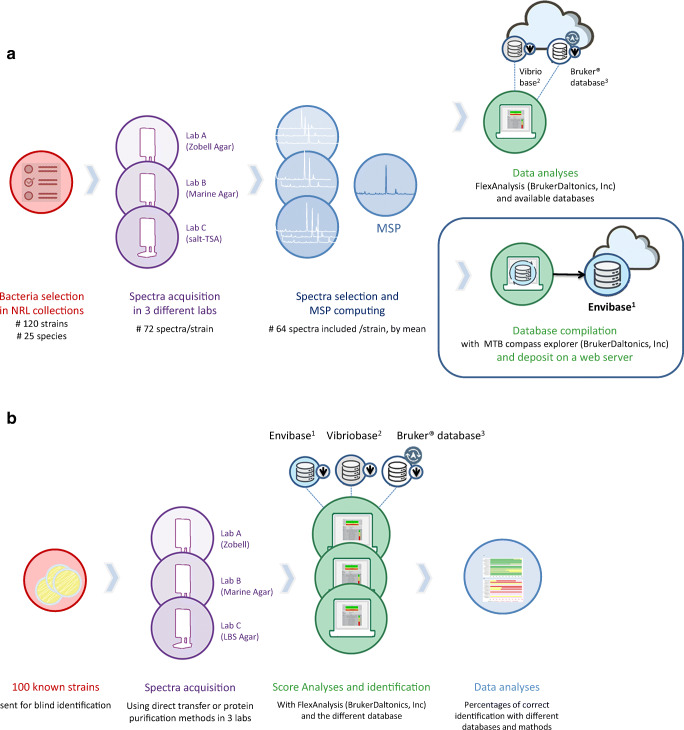
Main steps of EnviBase creation (**a**) and validation (**b**). ^1^Free download on 10.17882/75416. ^2^Erler et al. 2015, available on demand. ^3^Need subscription for downloading and annual updates (Bruker Daltonics, Inc.)

Each strain was grown in three different media: Zobell agar (peptone 4 g/L, yeast extract 1 g/L, Tris buffer 0.5 g/L in artificial seawater), Marine agar (Conda), and 1.5% salted TSA agar (Sigma-Aldrich) used respectively by Labeo-Manche, Qualyse, and Labocea labs collaborators (Fig. 1a). The incubation temperature was performed at 20 and 22°C during 48 h except for *V. aestuarianus* (72h).

### Sample preparation for MALDI-TOF main spectra (MS) analysis

#### Protein extraction

One loop of biomass from each culture was suspended in 300 μL HPLC (high performance liquid chromatography) water. A biomass calibration at 4–5 McFarland was realized. A volume of 900 μL pure ethanol was added to sterilize the bacteria and denature the proteins. The supernatant was discarded, and the pellet was centrifuged again to remove ethanol residues and dried at room temperature. Thirty microliters of formic acid 70% was added followed by the same volume of acetonitrile. After centrifugation, 1 μL of supernatant was spotted onto a MALDI 96 MSP target polished steel # 8280800in eight replicates and overlaid with 1 μL HCCA matrix solution (α-Cyano-4-hydroxycinnamic acid, # 8255344, Bruker).

### Spectrum acquisition—mass spectrometry measurement

Spectra were acquired using the Microflex LT/SH system (Bruker Daltonics Inc., Bremen). Each spot was analyzed three times, resulting in 24 single spectra per medium for each strain. A total of 72 spectra were generated for each strain.

As Bruker recommended, a Bacterial Test Standard (BTS 8255343) was used to calibrate the instrument, before each acquisition session. A BTS was also used on each acquisition plate, to check the quality of the acquisition. As the most distinct, clear, and significant spectra lie in a mass range of 5000 to 10,000 Da, the protein mass spectra of samples were acquired in a mass range of 2000–20,000 Da by the flexControl 3.4 program (Bruker Daltonics). The laser frequency for every run was 60 Hz with a linear positive mode. The default settings of the MALDI-TOF MS instrument were as follows: lens, 8.5 kV; ion source 1, 20 kV; and ion source 2, 18.1 kV. The flexControl program automatically acquired the spectrum of each spot. Each spectrum was generated by 240 laser shots (40 laser shot steps at six randomly selected positions of a single spot).

### *Vibrio* database development—software-based analyses

To create a main spectrum projection (MSP) corresponding to a reference spectrum affiliated to each strain, the raw spectra were analyzed one by one to identify nonspecific spectra. First, using the flexAnalysis software (version 3.4, Bruker Daltonics, Inc.), the main spectral features and shape were analyzed according to the recommendations of Bruker Daltonics (MALDI Biotyper^R^ V1.1), and low quality spectra (spectra with outlier peaks and flatlines, shift spectra) were excluded. The results of the visual inspection method were further confirmed by two software-based analyses: cluster analysis (PCA) and composite correlation index (CCI) matrix analysis (MALDI Biotyper Compass Explorer module v4.1).

The dataset consisted of mass spectra obtained from the analyzed strains for which the intensities of the mass to charge ratios (m/z) constituted the variables. The PCA clustering statistical analysis allowed a visualization of the relationships between the spectra highlighting the different groups and outliers by reducing the dataset (MALDI Biotyper Compass User).

A CCI matrix analysis was also performed to study the relationships between the spectra, indicating the statistical distance between them. The acquired spectra were subsequently analyzed by the tool in the MALDI Biotyper Compass Explorer software to determine the CCI, a parameter to estimate the distance between spectra. A CCI match value of 1 represents complete correlation, whereas a CCI match value of 0 represents an absence of correlation. The numerical CCI values were automatically visualized as a “heatmap” in the CCI matrix window. The closeness of the spectra was indicated by the color of the squares on the heatmap. Red color indicates closely related spectra; blue color indicates that they are not closely related.

Then, for each strain, the spectra selected by the previously described methods were merged using the MALDI Biotyper Compass Explorer software (version 4.1.90) to generate a main reference spectrum (MSP). Hence, an MSP was created from the compilation of the spectra generated in three different media for each strain (by three different laboratories) to provide diversity of conditions, which demonstrate the robustness of the results obtained. The new EnviBase was compiled based on 120 created MSP. Finally, these newly created MSP were tested by comparing matching scores obtained with Bruker’s and Erler’s databases (Erler et al. 2015), which contain7854 and 997 MSP, respectively (Fig 1a).

#### Dendrogram generation

The classification of all strains was verified by creating an MSP dendrogram using the MALDI Biotyper Compass Explorer software (version 4.1.90).

### MALDI-TOF MS *Vibrio* database validation

To validate the EnviBase, a blind test was performed by testing 100 strains by the three collaborating laboratories (Fig. 1b). Table S3 presents a description of the strains used for this test.

Two different methods were used in order to assess their performance in routine use on the results: the whole protein extraction technique (protocol above) and the direct transfer technique. For the latter technique, one loop of a fresh bacteria colony was directly smeared onto a MALDI target plate (96 MSP target polished steel #8280800) and covered with saturated HCCA matrix solution (alpha-cyano-4-hydroxycinnamic acid dissolved in 50% acetonitrile, 47.5% LC-MS water and 2.5% trifluoroacetic).

These strains were analyzed by MALDI-TOF MS, and the raw spectra generated were first compared with the newly created *Vibrio* database alone, and then with the three grouped databases (Bruker, Erler and our EnviBase) (Fig. 1b). The results were indicated by a logarithmic score. The matching scores for the reference main spectra were divided into ranges reflecting highly probable species identification (>2.3), secure genus identification and probable species identification (2.0–2.299), probable genus identification (1.7–1.999), and unreliable identifications (<1.7).

#### Statistical analysis

The percentage of the number of well-identified strains was calculated for each sample protocol technique. The two methods were compared using the non-parametric Wilcoxon rank test for paired series with the XLSTAT software (Add in soft). *p*-value*s* < 0.05 were considered to show statistically significant difference between the 2 results.

## Results

### The MALDI-TOF spectra selection method for bacterial species differentiation

All of the acquired spectra (24 spectra per strain per laboratory, giving a total of 72 spectra per strain) were analyzed with different methods described above (Visual and statistical methods), in order to select the most representative spectra and merge them into strain-specific MSP, which were used as the reference spectra.

**Fig. 2:**
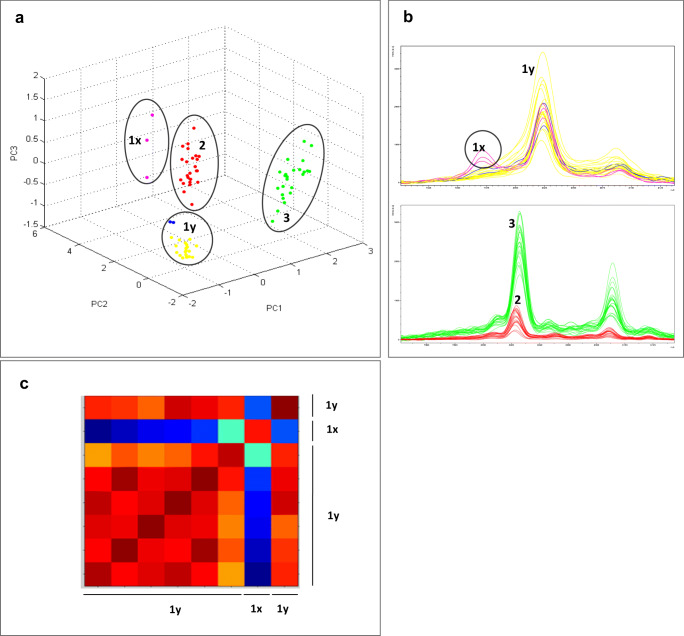
Raw spectral analysis using three different methods on the strain *Vibrio bivalvicida* CECT 8856. **a** PCA cluster analysis. Classification of the proteomic mass spectra of the strain cultivated in three different media in the first three principal components (PC1, PC2, PC3) using MALDI Biotyper Compass Explorer software. 1x and 1y: 1.5% salted TSA medium. 2: Zobell medium. 3: marine agar medium. **b** Raw spectra. Visualization of the spectra using the flexAnalysis software. **c** CCI matrix. CCI values obtained using spectra from group 1. Cold colors indicate weakly correlated spectra, and warm colors indicate highly correlated spectra

First, the spectra were selected according to Bruker’s recommendations by examining the peaks. Then, a three-dimensional principal component analysis (3D-PCA) scatter plot was used as it clearly showed distinguishable clusters and made it possible to remove the spectra that were the most discordant, i.e., outliers. For instance, independent of any effect caused by the medium, certain spectra for one strain (*V. bivalvicida*) plotted away from the rest of the 24*3 generated spectra (Fig. 2a; spectra groups 1x and 1y). The flexAnalysis software was used to observe these spectra and showed that they were different from the others, including spectra within the same group (Fig. 2b). Finally, the CCI matrix analysis confirmed the exclusion of spectra in cluster 1x (Fig. 2c). Using these three tools, divergent spectra were discarded, and representative spectra were selected for each strain and each laboratory and were then merged into the MSP, each of which contains by mean 64 selected spectra (min 39, max 72 selected spectra).

**Fig. 3:**
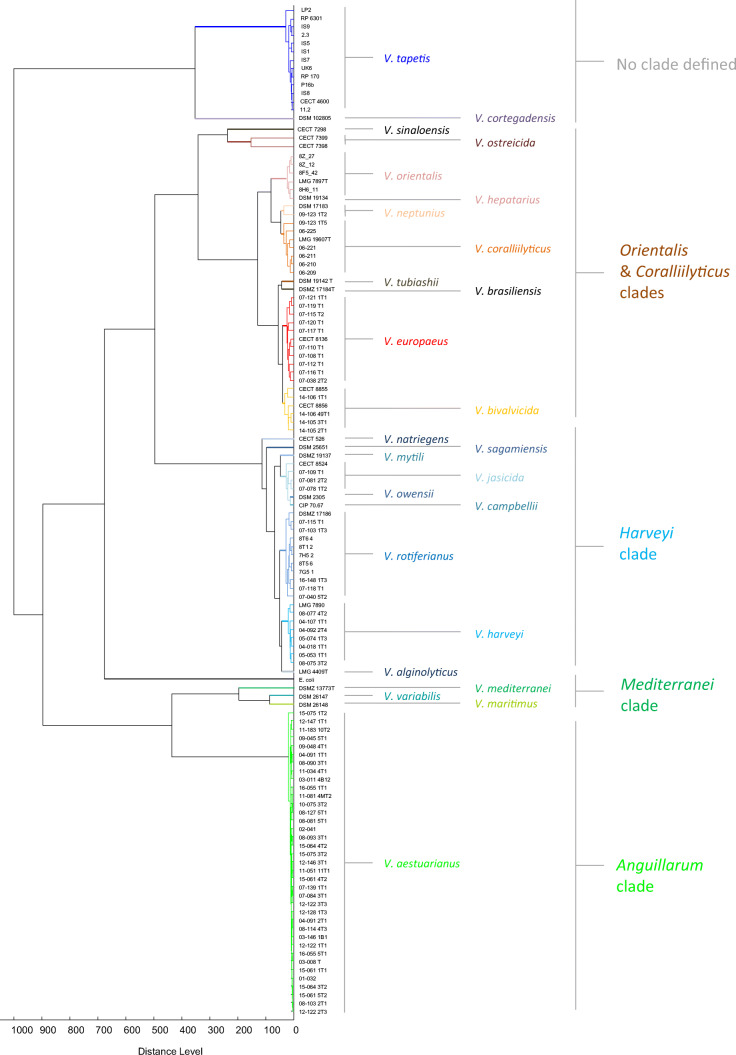
Dendrogram generated using Bruker’s MBT Compass Explorer based on MSP spectra. Species affiliations are indicated by the colors. Genetically defined clade affiliations are also indicated on the right-hand side

Bacterial isolates belonging to the same species were found to be grouped within the same clade on the MSP dendrogram (Fig. 3), in accordance with the *Vibrio* clades previously defined using genetic tools (Sawabe et al. 2007, 2013). However, one exception concerns the *Coralliilyticus* clade, which formed a cluster with part of *Orientalis* clade including *V. orientalis*, *V. sinaloensis*, and *V. hepatarius* as well as with *V. europaeus* and *V. bivalvicida* (Fig. 3).

### Bacterial identification with MALDI-TOF MS is dependent on the database queried

Within the 25 species included in our database and selected as marine mollusk pathogens, 12 are already present in the Bruker^TM^ database, and six can be found in the VibrioBase (Erler et al. 2015) but sometimes with only one representative strain (Table 1). In our mollusk pathogen VibrioBase (EnviBase), we included new isolates, especially “clinical” strains isolated from mollusk mortality episodes, such as *V. aestuarianus*, *V. coralliilyticus*, and *V. rotiferianus* (Table 1).

**Table 1:** Numbers of main spectra projections (MSP) for the *Vibrio* species that are potentially pathogenic for marine mollusks filed in the Bruker^TM^ database, VibrioBase (Erler et al. 2015), and our new mollusk pathogen database (EnviBase)

MALDI-TOF MS species	Bruker^TM^	VibrioBase	EnviBase
	MSP number	MSP number	MSP number
*V. aestuarianus*	4	7	37
*V. alginolyticus*	5	255	1
*V. bivalvicida*	0	0	6
*V. brasiliensis*	0	0	1
*V. campbellii*	1	0	1
*V. coralliilyticus*	1	1	7
*V. cortegadensis*	0	0	1
*V. europaeus*	0	0	11
*V. harveyi*	7	46	8
*V. hepatarius*	0	0	1
*V. jasicida*	0	0	4
*V. maritimus*	0	0	1
*V. mediterranei*	1	1	1
*V. mytili*	1	0	1
*V. natriegens*	2	1	1
*V. neptunius*	1	0	2
*V. orientalis*	1	0	5
*V. ostreicida*	3	0	2
*V. rotiferianus*	1	0	11
*V. sagamiensis*	0	0	1
*V. sinaloensis*	0	0	1
*V. tapetis*	0	0	13
*V. tubiashii*	0	0	1
*V. owensii*	0	0	1
*V. variabilis*	0	0	1
Total	28	311	120

After the generation of 120 new MSP for these 25 *Vibrio* species (Fig. 1a, Supplementary Table S1), we first tested them among the two available databases. Overall, using these databases, only 62% (*n*=74/120) of the MSP gave correct identifications (Supplementary Table S2).

**Fig. 4:**
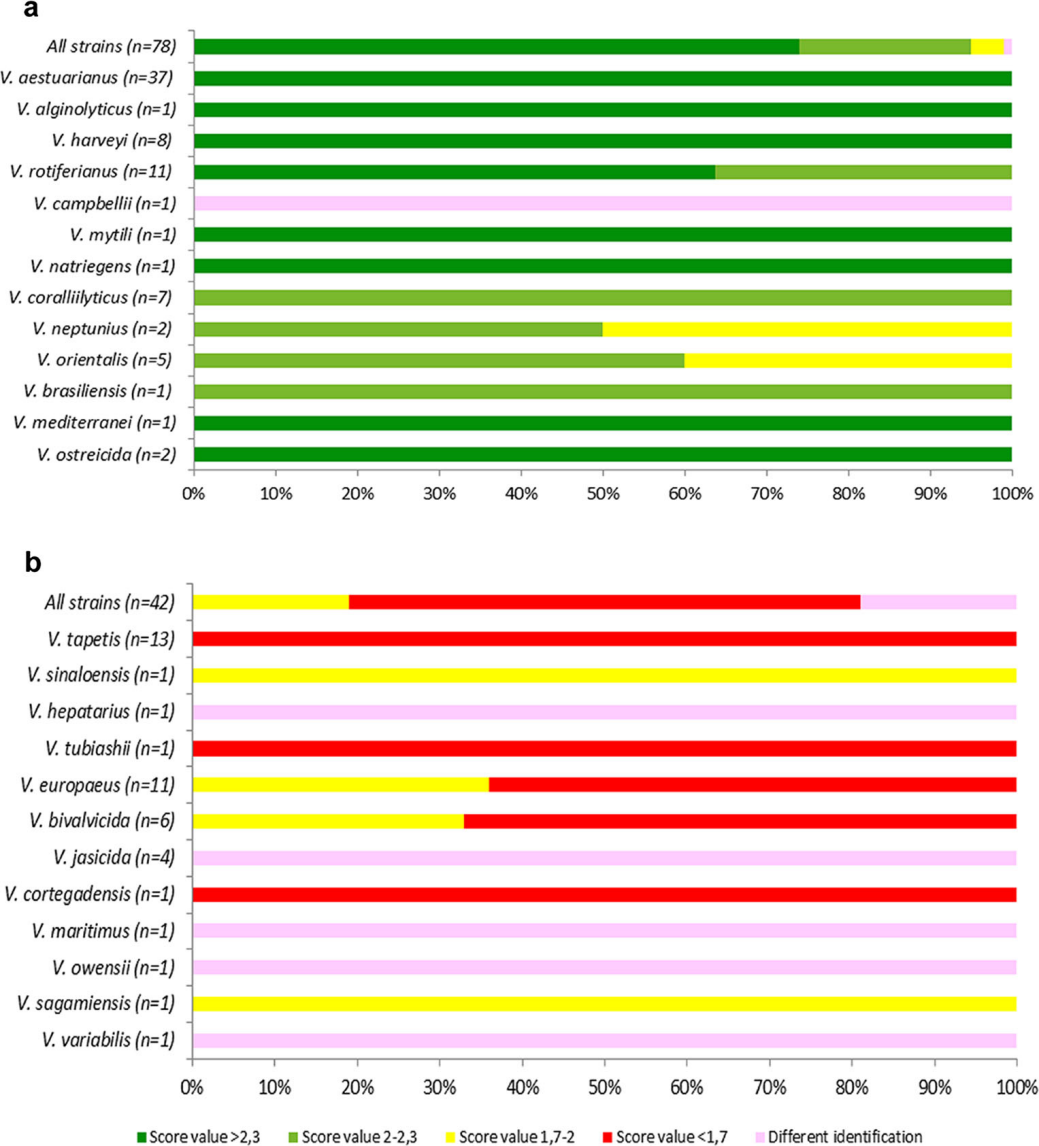
Identification of the new main reference spectra (MSP) with the Bruker^TM^ database and Erler’s database. **a** Only the MSP of the *Vibrio* species present in both databases were tested. **b** *Vibrio* species not present in either database. The highest matching scores of the new main reference spectra were represented by ranges indicating highly probable species identifications (≥2.3), probable species identifications (2.0–2.299), secure genus identifications (1.7–1.999), and unreliable identifications (<1.7). Assignment of strains obtained with Bruker^TM^ database that were different from the assignment of species of EnviBase main spectra were classified as “different identification”

However, when considering *Vibrio* species that were present in at least one of the two databases (the Bruker database and Erler’s database), 95% of our new MSP (*n*=74) were well-identified, among which we find *V. aestuarianus*, *V. harveyi*, *V. rotiferianus*, *V. coralliilyticus*, *V. alginolyticus*, *V. mediterranei*, *V. ostreicida*, *V. mytili*, *V. natriegens*, and *V. brasiliensis* (Fig. 4a, Supplementary Table S2). In particular, 74% of the MSP created had a matching score over 2.3 (highly probable species identification), and 21% had a score between 2.0 and 2.3 (probable species identification). It is important to note that 4% of the MSP had a score value between 1.7 and 2.0 for *V. neptunius* and *V. orientalis* and 1% gave a different identification for *V. campbellii* despite these species being given in the Bruker database (Fig. 4a, Supplementary Table S2). The identities of the strains of *V. neptunius* (09/123 1T2; DSM 17183), *V. orientalis* (DSM 7897T; 8F5_42; 8H6_11; 8Z_12; 8Z_27), and *V. campbellii* (CIP 70.67) we used were confirmed by PCR and DNA sequencing (data not shown).

Moreover, as expected, all of the strains affiliated with species that are not present in the existing databases had a score below 2.0 or were classified under a different identification. The mismatches between these species and Bruker’s and Erler’s databases show the input of the newly created MSP included in EnviBase. For instance, *V. bivalvicida*, *V. europaeus*, *V. sagamiensis*, *V. sinaloensis*, *V. tapetis*, and that the *V. tubiashii* strains had a score below 1.7 with Bruker’s and Erler’s databasesand *Vibrio hepatarius*, *V. maritimus*, *V. variabilis*, *V. jasicida*, and *V. owensii* were classified within different species (*V. orientalis*, *V. mediterranei*, *V. mediterranei*, *V. harveyi*, and *V. harveyi*, respectively) (Fig. 4b, Supplementary Table S2). In cases where a species had a different identification, it should be noted that the databases recognized the closest species, or at least a species defined within the same clade.

### MALDI-TOF MS EnviBase validation through a blind test

**Fig. 5:**
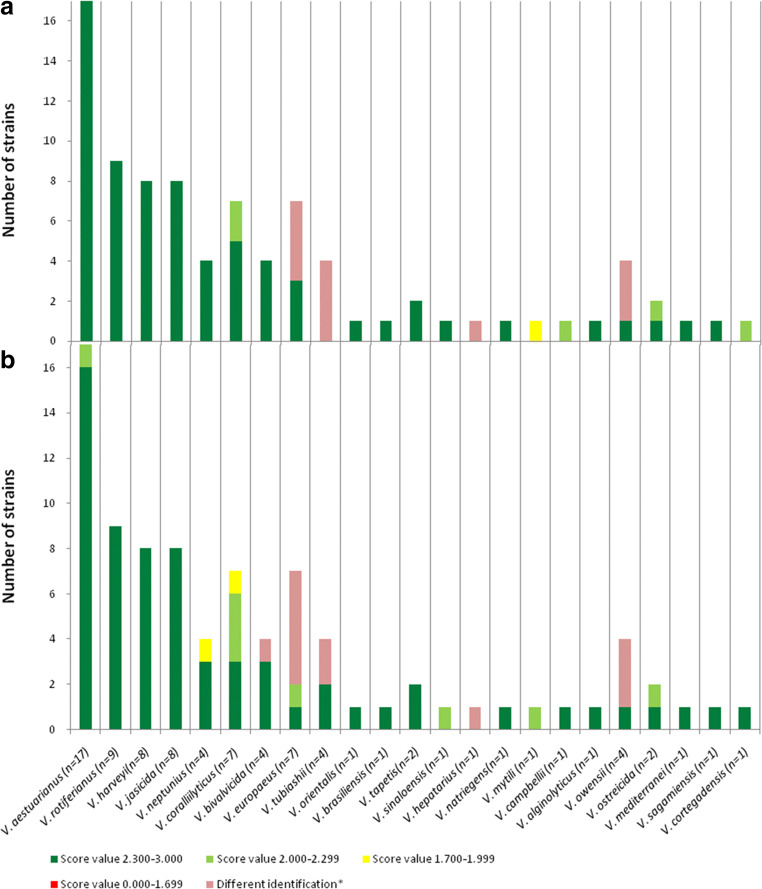
Blind test on the *Vibrio* sp. present in the database identified by MALDI-TOF MS with the new created EnviBase performed by one laboratory using the (**a**) direct transfer and (**b**) protein extract methods. Different colors are used to indicate the score values

In a blind test performed on 100 strains (Fig 1b), 87%, 83%, and 84% of the *Vibrio* species tested with the newly created EnviBase by the three collaborating laboratories were correctly identified with the direct transfer method (Fig. 5a). These percentages reached 87%, 83%, and 86% correct identifications with the protein extract method (Fig. 5b, Table S4A). There are no significant differences between the two methods according to the Wilcoxon test (*p*-value = 1).

A small percentage of the results for the direct transfer and protein extraction methods performed on *Vibrio* species by the three laboratories were considered to be doubtful (12%, 13%, and 6% versus 12%, 13%, and 12%, respectively) because they showed an initial score above 2.0 for the correct identifications; however, the nine following results showed very similar scores close to 2.0 either with an incorrect identification or the first score was obtained with an incorrect identification followed very closely by the correct identification, making it impossible to discern between two very similar species. This is the case for *V. europaeus*/*V. tubiashii*/*V. bivalvicida*, *V. hepatarius*/*V. orientalis*, and *V. owensii/V. jasicida/V. campbellii*.

A very small percentage of the scores determined using the direct transfer and protein extraction methods (1%, 1%, 6%, versus 2%, 1%, and 1%, respectively) performed on the *Vibrio* species could be used to verify only the genus level alone (1.700–1.999). There were 0%, 2%, and 2% misidentifications with a matching score above 2.00 for the first method and 0%, 3%, and 0% for the second. Very closely related species belonging to *V. tubiashii*/*V. europaeus* and *V. owensii*/*V. jasicida*/*V. campbellii* (Table S4A) were incorrectly identified but still had a high matching score with the two methods. Two percent (2%) of the species obtained a score below 1.7 with the protein extract method.

For all of the tested species not present in the newly created database (*n*=13) and for the three laboratories, some species were not identified at all or had a different identification, showing the specificity of our database (Table S4B).

The blind test performed with the three databases (EnviBase, Bruker’s, and Erler’s databases), revealed that the best matching scores were obtained with our new database, with an average rate of 98% for all of the strains found in all of the databases (52/53) for both methods (data not shown). However, we did observe some misidentifications for the *V. jasicida* strains when tested with the compiled databases. These were identified as *V. harveyi* with Bruker’s and Erler’s databases.

### Database download

**Fig. 6:**
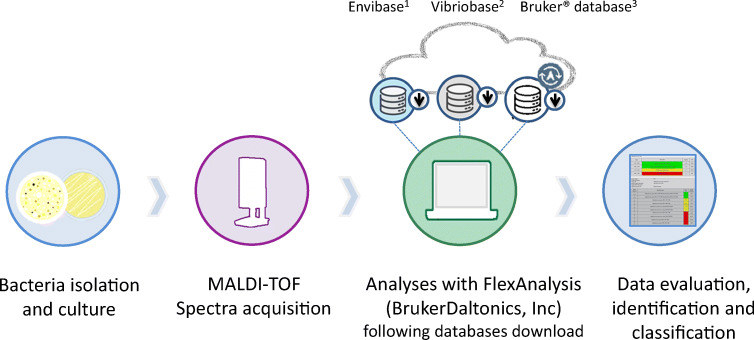
MALDI-TOF MS microbial identification workflow. ^1^Free download on 10.17882/75416. ^2^Erler et al. 2015, available on demand. ^3^Need subscription for downloading and annual updates (Bruker Daltonics, Inc.)

The EnviBase could be downloaded on SEANOE (Sea scientific open data edition) as a btmsp file on the following link 10.17882/75416. All MSP of the database have to be imported into MBT compass software to be queried for identification (Fig. 6).

## Discussion

In this study, for the first time, a MALDI-TOF MS database was built collecting most of the *Vibrio* species involved in mollusk diseases. The EnviBase comprised 120 MSP containing 25 *Vibrio* species including 12 species that are currently absent from the other existing databases. This database was tested face to Bruker Daltonics database and the VibrioBase (Erler et al. 2015). Another MALDI-TOF MS database called EMbaRC has been recently developed and includes 77 spectra of bacteria of *Vibrionaceae* family (Ruvira et al. 2013), but as this database mostly contains *Vibrio* type strains already available in Bruker’s one, it was not included in this study.

### Spectrum selection and MSP creation

In order to integrate the most specific MSP in the database, we defined a pipeline for spectrum selection via the different analysis tools. In the conventional spectrum selection method, the analysis focuses on peaks that have the highest intensity within the range of mass peaks from 3000 and 12,000 Da (MALDI Biotyper V1.1 protocol). However, peaks with a lower intensity could reveal significant differences between isolates. These differences could have an impact on the specificity of the spectra, especially in very closely related species. Thus, we used a principal component analysis (PCA) (Shao et al. 2012; Vallenet et al. 2019) and the composite correlation index (CCI matrix) (Arnold and Reilly 1998; Carrasco et al. 2014; Magalhães et al. 2018) to identify the spectra that were not specific enough due to these differences. Our data processing allowed to create more specific MSP and thus to discriminate between very close species such as *V. bivalvicida*/*V. europaeus* or *V. neptunius*/*V. coralliilyticus*. In general, the PCA clusters and CCI matrix were used to visualize the relationships between the spectra (Shao et al. 2012). Hence, the application of this statistical method to select spectra could confirm the reproducibility of the spectra for the same strain in the same conditions (e.g., for one laboratory).

The procedure we recommend first consists of analyzing the spectra using flexAnalysis software to check the intensity of the peaks and the general shape of the spectra. In a second step, the spectra are loaded into Bruker’s MBT Compass software where they are then identified one by one using existing databases to eliminate mismatched spectra. Third, PCA clustering and the CCI matrix are used to confirm the exclusion of spectra or to exclude spectra that are insufficiently specific and for which the differences are not easily detectable using flexAnalysis.

### Database assessment

The new EnviBase was evaluated by testing the created MSP with the existing databases. Considering the species present in these databases, the majority of species were correctly identified except for certain strains like *V. campbellii*, which was indistinguishable from the closely related *V. harveyi*. Specific effort is needed to build highly specific MSP that can be used to discriminate between these very similar species (Azevedo et al. 2003; De Montaudouin et al. 2009; Gomez-Gil et al. 2004).

Most of the other strains belonging to species present in Bruker’s database were correctly identified, although some low scores were observed for certain species such as the *V. neptunius* and *V. orientalis* strains (between 1.7 and 2). These low scores could be explained by a poor representativeness of the bacterial species in the database (e.g., there is only one strain each for *V. neptunius* and *V. orientalis* in the Bruker database). Erler et al. (2015) showed the importance of having a sufficient number of strains for a given species in order to encompass its intra-specific diversity. Future updates of the databases with diverse strains for each species could improve the identification of any given species.

### Validation of the database

After the EnviBase assessment, we carried out a validation procedure with a blind test performed with two MALDI-TOF analysis methods: direct transfer (usually used in routine diagnoses) and whole protein extraction. No significant differences were observed between the two extraction methods with regard to species identification. The only differences observed were differences in the identification rates between the two methods for certain species. The structure of the bacteria probably has an influence on the accuracy of their identification (Eck and Dayhoff 1966). Meanwhile, the direct transfer method is faster to implement and is, therefore, more suitable for routine diagnosis. Consequently, this method can be used and is even recommended in routine diagnoses.

Some species, such as *V. europaeus*/*V. tubiashii*, were misidentified during the blind test given their high taxonomic proximity. It should be noted that *V. europaeus* and *V. tubiashii* have undergone numerous taxonomic revisions: *V. europaeus* was considered to be a sub-species of *V. tubiashii* before being reclassified as a distinct species (Dubert et al. 2016).

For other species, no conclusion could be drawn as to why *V. jasicida* was identified as *V. harveyi* when the databases were pooled. This is likely linked to the fact that *V. jasicida* is not represented in Bruker’s or Erler’s databases or, possibly, that it has been incorrectly affiliated with some of the MSP for *V. harveyi* present in these databases. The difficulties to differentiate and characterize *Harveyi* clade species due to their taxonomic proximity were illustrated recently by Ke et al. (2017) who showed that a *V. harveyi* 1114GL strain affecting shrimp should be in fact affiliated with the *V. campbellii* species. The high similarity in the rDNA sequences and phenotypes made it risky to identify these species (Ezaki et al. 1989; Yoshizawa et al. 2012). Furthermore, we encountered difficulties when attempting to discriminate *V. owensii* from *V. jasicida* as there is only one representative strain for the latter. This should be improved in future work.

The majority of the sub-species were correctly identified (data not shown), but even if we do not have clear identifications, the MALDI-TOF MS technique could be sufficient enough to identify the correct sub-species if the specificity of the infected host is taken into consideration. Three sub-species have been described for *V. aestuarianus aestuarianus*, *V. aestuarianus francensis* associated with cupped oyster diseases, and *V. aestuarianus cardii*, associated with cockle diseases (Garcia et al. 2021). Further analysis could be applied to sub-type species or to discriminate between pathogenic and non-pathogenic strains, e.g., the “MALDI-TOF-MS Multi Peak Shift Typing approach,” highlighting a link between a peak shift in the mass spectra and the associated polymorphism in the genomic sequence (Bridel et al. 2020; Rahmani et al. 2021).

The combination of the MALDI-TOF MS technique with this new database is a powerful and revolutionary method within the context of surveillance programs and emerging diseases. Our database could also be used as a potent molecular epidemiologic tool during outbreaks (Taneja et al. 2016). In the present work, we validate the direct transfer MALDI sample preparation method which is a real advantage in monitoring programs.

In the future, we intend to expand this database by adding species of the fundamental and highly diverse *Splendidus* clade, which is associated with significant mortality events (Bruto et al. 2017; Faury et al. 2004; Gay et al. 2004; Le Roux 2017; Lemire et al. 2015; Petton et al. 2015).

## Supplementary information

ESM 1 (PDF 1066 kb).

## Data Availability

All of the MSP in the EnviBase are freely accessible as a btmsp file on link 10.17882/75416.
