# Supplementary material for: A MALDI-TOF MS database for fast identification of Vibrio spp. potentially pathogenic to marine mollusks
Source: Appl Microbiol Biotechnol. 2021 Feb 15;105(6):2527–39. doi: 10.1007/s00253-021-11141-0 (PMC7954726; doi:10.1007/s00253-021-11141-0)
Supplement: Supplementary file 1 — (PDF 1066 kb). [file 253_2021_11141_MOESM1_ESM.pdf]

Applied Microbiology and Biotechnology

A MALDI-TOF MS database for fast identification of *Vibrio* spp. potentially pathogenic to marine mollusks

M. Moussa[a], E. Cauvin[c], A. Le Piouffle[d], O. Lucas[e], A. Bidault[f], C. Paillard[f], F. Benoit[c], B. Thuillier[d], M. Treilles[e], M. A. Travers[a, b*], C. Garcia[a#*]

[a]Laboratoire de Génétique et Pathologie des Mollusques Marins, SG2M-LGPMM, Ifremer, F-17390 La Tremblade, France

[b]IHPE, Univ. Montpellier, CNRS, Ifremer, Univ. Perpignan Via Domitia. F-34090 Montpellier, France

[c]Labeo-Manche, 1352 avenue de Paris 50000 Saint-Lô, France

[d]Labocea, Avenue de la Plage des Gueux 29330 Quimper, France

[e]Qualyse, ZI Montplaisir, 79220 Champdeniers Saint-Denis, France

[f]Univ Brest, CNRS, IRD, Ifremer, UMR6539 LEMAR, F-29280 Plouzané, France

Table S1. 120 Vibrio strains used for MSP creation, included in the ~~MP~~ EnviVibrioBase.

Clade	Species	Strain number	Sampling date	Sampling location	Sample origin
-	***V. cortegadensis***	**DSM 102805T**	2005	Spain	Manila clam,*Ruditapes philippinarum*
-	*V. tapetis tapetis*	IS1	1988	Landeda (France)	Manila clam,*Ruditapes philippinarum*
-	*V. tapetis tapetis*	IS9	1990	Quiberon (France)	Cockle, *Cerastoderma edule*
-	*V. tapetis tapetis*	IS8	1990	Quiberon (France)	Clam, *Polititapes aureus*
-	*V. tapetis tapetis*	IS7	1990	Quiberon (France)	Manila clam,*Ruditapes philippinarum*
-	*V. tapetis tapetis*	IS5	1991	Landeda (France)	Manila clam,*Ruditapes philippinarum*
-	*V. tapetis tapetis*	RP 6301	1992	Spain	Manila clam,*Ruditapes philippinarum*
-	*V. tapetis tapetis*	RP 1703	1994	Spain	Manila clam,*Ruditapes philippinarum*
-	*V. tapetis tapetis*	P16b	1995	Golfe du Morbihan, France	Manila clam,*Ruditapes philippinarum*
-	*V. tapetis tapetis*	LP2	1999	Norway	Corkwing Wrasse,*Symphodus melops*
-	*V. tapetis tapetis*	UK6	1997	United Kingdom	Manila clam,*Ruditapes philippinarum*
-	***V. tapetis tapetis***	**CECT 4600T**	1990	Landeda (France)	Manila clam,*Ruditapes philippinarum*
-	*V. tapetis tapetis*	2.3	1990	Landeda (France)	Manila clam,*Ruditapes philippinarum*
-	*V. tapetis tapetis*	11.2	1990	Landeda (France)	Manila clam,*Ruditapes philippinarum*
Anguillarum	*Vibrio aestuarianus francensis*	04-091_1T1	2004	Finistère (France)	Flat oyster, *Ostrea edulis*
Anguillarum	*Vibrio aestuarianus francensis*	04-091_2T1	2004	Finistère (France)	Flat oyster, *Ostrea edulis*
Anguillarum	*V. aestuarianus*	16-055_5T1	2016	Morbihan (France)	Mussel*, Mytilus sp.*
Anguillarum	*V. aestuarianus*	16-055_1T1	2016	Morbihan (France)	Mussel, *Mytilus sp.*
Anguillarum	***Vibrio aestuarianus aestuarianus***	**LMG 7909T**	Before 1985	USA	Oyster
Anguillarum	*Vibrio aestuarianus cardii*	12-122 1T1 (LMG 31437=DSM 109724)	2012	Baie de Somme (France)	Cockle, *Cerastoderma edule*
Anguillarum	*Vibrio aestuarianus cardii*	12-122_2T3	2012	Baie de Somme (France)	Cockle, *Cerastoderma edule*
Anguillarum	***Vibrio aestuarianus cardii***	**12-122_3T3 (LMG 31436T=DSM 109723T)**	2012	Baie de Somme (France)	Cockle, *Cerastoderma edule*
Anguillarum	*Vibrio aestuarianus cardii*	15-075_3T2(LMG 31440=DSM 109720)	2015	Baie de Somme (France)	Cockle, *Cerastoderma edule*
Anguillarum	*Vibrio aestuarianus cardii*	15-061_1T1	2015	Baie de Veys (France)	Cockle, *Cerastoderma edule*
Anguillarum	*Vibrio aestuarianus cardii*	15-061_4T2(LMG 31438=DSM 109725)	2015	Baie de Veys (France)	Cockle, *Cerastoderma edule*
Anguillarum	*Vibrio aestuarianus cardii*	15-061_5T2	2015	Baie de Veys (France)	Cockle, *Cerastoderma edule*
Anguillarum	*Vibrio aestuarianus cardii*	15-075_1T2	2015	Baie de Somme (France)	Cockle, *Cerastoderma edule*
Anguillarum	*Vibrio aestuarianus cardii*	15-064_3T2(LMG 31439=DSM 109719)	2015	Binic (France)	Cockle, *Cerastoderma edule*
Anguillarum	*Vibrio aestuarianus cardii*	15-064_4T2	2015	Binic (France)	Cockle, *Cerastoderma edule*
Anguillarum	***Vibrio aestuarianus francensis***	**02-041 (LMG 24517T = CIP 109791T)**	2002	Brittany (France)	Pacific oyster, *Crassostrea gigas*
Anguillarum	*Vibrio aestuarianus francensis*	03-011-4B12	2003	Charente-Maritime (France)	Pacific oyster, *Crassostrea gigas*
Anguillarum	*Vibrio aestuarianus francensis*	03-146-1B1	2003	Charente-Maritime (France)	Pacific oyster, *Crassostrea gigas*

Anguillarum	*Vibrio aestuarianus francensis*	07-139-1T1	2007	Calvados (France)	Pacific oyster, *Crassostrea gigas*
Anguillarum	*Vibrio aestuarianus francensis*	07-084-3T1	2007	Charente-Maritime (France)	Pacific oyster, *Crassostrea gigas*
Anguillarum	*Vibrio aestuarianus francensis*	08-090-3T1	2008	Charente-Maritime (France)	Pacific oyster, *Crassostrea gigas*
Anguillarum	*Vibrio aestuarianus francensis*	08-093-3T1	2008	Vendée (France)	Pacific oyster, *Crassostrea gigas*
Anguillarum	*Vibrio aestuarianus francensis*	08-127-5T1	2008	Morbihan (France)	Pacific oyster, *Crassostrea gigas*
Anguillarum	*Vibrio aestuarianus francensis*	08-103-2T1	2008	Morbihan (France)	Pacific oyster, *Crassostrea gigas*
Anguillarum	*Vibrio aestuarianus francensis*	08-081-5T1	2008	Charente-Maritime (France)	Pacific oyster, *Crassostrea gigas*
Anguillarum	*Vibrio aestuarianus francensis*	08-114-4T3	2008	Manche (France)	Pacific oyster, *Crassostrea gigas*
Anguillarum	*Vibrio aestuarianus francensis*	11-081-4MT2	2008	Calvados (France)	Pacific oyster, *Crassostrea gigas*
Anguillarum	*Vibrio aestuarianus francensis*	09-045-5T1	2009	Finistère (France)	Pacific oyster, *Crassostrea gigas*
Anguillarum	*Vibrio aestuarianus francensis*	09-048-4T1	2009	Gironde (France)	Pacific oyster, *Crassostrea gigas*
Anguillarum	*Vibrio aestuarianus francensis*	10-075-3T2	2010	Charente-Maritime (France)	Pacific oyster, *Crassostrea gigas*
Anguillarum	*Vibrio aestuarianus francensis*	11-183-10T2	2011	Corse (France)	Pacific oyster, *Crassostrea gigas*
Anguillarum	*Vibrio aestuarianus francensis*	01-032	2011	Brittany (France)	Pacific oyster, *Crassostrea gigas*
Anguillarum	*Vibrio aestuarianus francensis*	11-034-4T1	2011	Hérault (France)	Pacific oyster, *Crassostrea gigas*
Anguillarum	*Vibrio aestuarianus francensis*	11-051-11T1	2011	Landes (France)	Pacific oyster, *Crassostrea gigas*
Anguillarum	*Vibrio aestuarianus francensis*	12-147-1T1	2012	Manche (France)	Pacific oyster, *Crassostrea gigas*
Anguillarum	*Vibrio aestuarianus francensis*	12-146-3T1	2012	Finistère (France)	Pacific oyster, *Crassostrea gigas*
Anguillarum	*Vibrio aestuarianus francensis*	12-128-1T3	2012	Finistère (France)	Pacific oyster, *Crassostrea gigas*
Coralliilyticus	*V. coralliilyticus*	06-209	2006	France	Pacific oyster, *Crassostrea gigas*
Coralliilyticus	*V. coralliilyticus*	06-210	2006	France	Pacific oyster, *Crassostrea gigas*
Coralliilyticus	*V. coralliilyticus*	06-211	2006	France	Pacific oyster, *Crassostrea gigas*
Coralliilyticus	*V. coralliilyticus*	06-221	2006	France	Pacific oyster, *Crassostrea gigas*
Coralliilyticus	*V. coralliilyticus*	06-225	2006	France	Pacific oyster, *Crassostrea gigas*
Coralliilyticus	***V. coralliilyticus***	**DSM 19607T**	1999	Indian Ocean	Corals, *Pocillopora damicornis*
Coralliilyticus	*V. coralliilyticus*	09-123_1T5	2009	Charente-Maritime (France)	Pacific oyster, *Crassostrea gigas*
Coralliilyticus	*V. neptunius*	09-123_1T2	2009	Charente-Maritime (France)	Pacific oyster, *Crassostrea gigas*
Coralliilyticus	***V. neptunius***	**DSM17183T**	1998	Brazil	Scallop, *Nodipecten nodosus*
Coralliilyticus	***V. ostreicida***	**CECT 7398T**	1999	Spain	Mollusc hatchery
Coralliilyticus	*V. ostreicida*	CECT 7399	1999	Spain	Mollusc hatchery
Harveyi	***V. alginolyticus***	**LMG 4409T**	Before 1973	Japan	Spoiled horse mackerel, *Trachurus trachurus*
Harveyi	*V. campbellii*	CIP 70.67	1968	Japan	Seawater
Harveyi	*V. harveyi*	08-077_4T2	2004	Hérault (France)	Pacific oyster, *Crassostrea gigas*
Harveyi	*V. harveyi*	04-018_1T1	2004	Manche (France)	Abalone*, Haliotis tuberculata*
Harveyi	*V. harveyi*	04-092_2T4	2004	Manche (France)	Abalone, *Haliotis tuberculata*
Harveyi	*V. harveyi*	04-107_1T1	2005	Manche (France)	Abalone, *Haliotis tuberculata*
Harveyi	*V. harveyi*	05-053_1T1	2005	Manche (France)	Abalone, *Haliotis tuberculata*

Harveyi	*V. harveyi*	05-074_1T3	2005	Manche (France)	Abalone, *Haliotis tuberculata*
Harveyi	*V. harveyi*	08-075_3T2	2008	Gironde (France)	Pacific oyster, *Crassostrea gigas*
Harveyi	*V. harveyi*	LMG 7890	1982	USA	Shark*, Carcharhinus plumbeus*
Harveyi	*V. jasicida*	CECT 8524	2004	Mexico	Fish, *Lutjanus guttatus*
Harveyi	*V. jasicida*	07-078_1T2	2007	Vendée (France)	Pacific oyster, *Crassostrea gigas*
Harveyi	*V. jasicida*	07-081_2T2	2007	Aude (France)	Pacific oyster, *Crassostrea gigas*
Harveyi	*V. jasicida*	07-109_T1	2007	Charente-Maritime (France)	Pacific oyster, *Crassostrea gigas*
Harveyi	***V. mytili***	**DSM 19137T**	Before 1985	Spain	Mussel*, Mytilus edulis*
Harveyi	***V. natriegens***	**CECT 526T**	1983	USA	Salt marsh mud
Harveyi	***V. owensii***	**DSM 23055T**	2007	Australia	Spiny lobster, *Panulirus ornatus larva*
Harveyi	***V. rotiferianus***	**DSM 17186T**	2003	Belgium	Rotifer, *Brachionus plicatilis*
Harveyi	*V. rotiferianus*	07-040_5T2	2007	Hérault (France)	Pacific oyster, *Crassostrea gigas*
Harveyi	*V. rotiferianus*	07-103_1T3	2007	Vendée (France)	Pacific oyster, *Crassostrea gigas*
Harveyi	*V. rotiferianus*	07-115_T1	2007	Charente-Maritime (France)	Pacific oyster, *Crassostrea gigas*
Harveyi	*V. rotiferianus*	07-118_T1	2007	Charente-Maritime (France)	Pacific oyster, *Crassostrea gigas*
Harveyi	*V. rotiferianus*	8T6_4	2014	Brittany (France)	Pacific oyster, *Crassostrea gigas*
Harveyi	*V. rotiferianus*	8T5_6	2014	Brittany (France)	Pacific oyster, *Crassostrea gigas*
Harveyi	*V. rotiferianus*	8T1_2	2014	Brittany (France)	Pacific oyster, *Crassostrea gigas*
Harveyi	*V. rotiferianus*	7H5_2	2014	Brittany (France)	Pacific oyster, *Crassostrea gigas*
Harveyi	*V. rotiferianus*	7G5_1	2014	Brittany (France)	Pacific oyster, *Crassostrea gigas*
Harveyi	*V. rotiferianus*	16-148_1T3	2016	Charente-Maritime (France)	Pacific oyster, *Crassostrea gigas*
Harveyi	***V. sagamiensis***	**DSM 25651T**	2008	Pacific Ocean equatorial region	Seawater
Harveyi	***V. sinaloensis***	**CECT 7298T**	2003	Mexico	Fish, *Lutjanus guttatus*
Mediterranei	***V. maritimus***	**DSM 26148T**	2005	Brazil	mucus of zoanthid, *Palythoa caribaeorum*
Mediterranei	*V. mediterranei*	DSM 13773	Before 1981	Spain	Marine sediment
Mediterranei	***V. variabilis***	**DSM 26147T**	2005	Brazil	mucus of zoanthid, *Palythoa caribaeorum*
Orientalis	*V. bivalvicida*	14-106_1T1	2014	Charente-Maritime (France)	Cockle, *Cerastoderma edule*
Orientalis	*V. bivalvicida*	14-106_49T1	2014	Charente-Maritime (France)	Cockle, *Cerastoderma edule*
Orientalis	*V. bivalvicida*	14-105_2T1	2014	Charente-Maritime (France)	Manila clam,*Ruditapes philippinarum*
Orientalis	*V. bivalvicida*	14-105_3T1	2014	Charente-Maritime (France)	Clam, *Ruditapes philippinarum*
Orientalis	***V. bivalvicida***	**CECT 8855T**	2008	Spain	Clam, *Ruditapes decussatus*
Orientalis	*V. bivalvicida*	CECT 8856	2008	Spain	Clam, *Ruditapes decussatus*
Orientalis	***V. brasiliensis***	**DSM 17184T**	2005	Brazil	Scallop,*Nodipecten nodosus*
Orientalis	*V. europaeus*	07-038_2T2	2007	Charente-Maritime (France)	Pacific oyster, *Crassostrea gigas*
Orientalis	*V. europaeus*	07-108_T1	2007	Charente-Maritime (France)	Pacific oyster, *Crassostrea gigas*
Orientalis	*V. europaeus*	07-110_T1	2007	Charente-Maritime (France)	Pacific oyster, *Crassostrea gigas*
Orientalis	*V. europaeus*	07-112_T1	2007	Charente-Maritime (France)	Pacific oyster, *Crassostrea gigas*

Orientalis	*V. europaeus*	07-115_T2	2007	Charente-Maritime (France)	Pacific oyster, *Crassostrea gigas*
Orientalis	*V. europaeus*	07-116_T1	2007	Charente-Maritime (France)	Pacific oyster, *Crassostrea gigas*
Orientalis	*V. europaeus*	07-117_T1	2007	Charente-Maritime (France)	Pacific oyster, *Crassostrea gigas*
Orientalis	*V. europaeus*	07-119_T1	2007	Charente-Maritime (France)	Pacific oyster, *Crassostrea gigas*
Orientalis	*V. europaeus*	07-120_T1	2007	Charente-Maritime (France)	Pacific oyster, *Crassostrea gigas*
Orientalis	*V. europaeus*	07-121_1T1	2007	Charente-Maritime (France)	Pacific oyster, *Crassostrea gigas*
Orientalis	***V. europaeus***	**CECT 8136T**	2001	Spain	Flat oyster, *Ostrea edulis*
Orientalis	***V. tubiashii***	**DSM19142T**	1991	USA	Clam, *Mercenaria mercenaria*
Orientalis	***V. hepatarius***	**DSM 19134T**	2007	Ecuador	Shrimps,*Litopenaeus vannamei*
Orientalis	***V. orientalis***	**DSM 7897T**	1984	Yellow Sea	Seawater
Orientalis	*V. orientalis*	8Z_27	2014	Brittany (France)	Pacific oyster, *Crassostrea gigas*
Orientalis	*V. orientalis*	8Z_12	2014	Brittany (France)	Pacific oyster, *Crassostrea gigas*
Orientalis	*V. orientalis*	8H6_11	2014	Brittany (France)	Pacific oyster, *Crassostrea gigas*
Orientalis	*V. orientalis*	8F5_42	2014	Brittany (France)	Pacific oyster, *Crassostrea gigas*

The names of the type strains are displayed in bold

Table S2. Best matching score values obtained for the 120 MSP in the ~~MP vibrio~~Envi~~b~~Base created with the Bruker database and Erler's database (**A**) for species present in both databases or in at least one database, (**B**) for species absent from these two databases.

A.

			**Bruker database**		**Erler database**	
**Clade**	**Species**	**Strain**	**Organism best match**	**Log score**	**Organism best match**	**Log score**
Anguillarum	*Vibrio aestuarianus francensis*	04-091_1T1	*Vibrio aestuarianus* DSM 19606	2.46	*Vibrio aestuarianus*VN-3828	2.55
Anguillarum	*Vibrio aestuarianus francensis*	04-091_2T1	*Vibrio aestuarianus* DSM 19606	2.45	*Vibrio aestuarianus*VN-3828	2.48
Anguillarum	*V. aestuarianus*	16-055_1T1	*Vibrio aestuarianus* VN_10100	2.51	*Vibrio aestuarianus*VN-3828	2.53
Anguillarum	*V. aestuarianus*	16-055_5T1	*Vibrio aestuarianus* VN_10100	2.57	*Vibrio aestuarianus*VN-3828	2.63
Anguillarum	***Vibrio aestuarianus aestuarianus***	**03-008T**	*Vibrio aestuarianus* VN_10100	2.59	*Vibrio aestuarianus*VN-3828	**2.62**
Anguillarum	*Vibrio aestuarianus cardii*	12-122_1T1	*Vibrio aestuarianus* VN_10100	2.52	*Vibrio aestuarianus*VN-3828	2.58
Anguillarum	*Vibrio aestuarianus cardii*	12-122_2T3	*Vibrio aestuarianus* VN_10100	2.52	*Vibrio aestuarianus*VN-3828	2.58
Anguillarum	*Vibrio aestuarianus cardii*	12-122_3T3	*Vibrio aestuarianus* DSM 19606	2.48	*Vibrio aestuarianus*VN-3828	2.56
Anguillarum	*Vibrio aestuarianus cardii*	15-061_1T1	*Vibrio aestuarianus* VN_10100	2.57	*Vibrio aestuarianus*VN-3828	2.66
Anguillarum	*Vibrio aestuarianus cardii*	15-061_4T2	*Vibrio aestuarianus* VN_10100	2.59	*Vibrio aestuarianus*VN-3828	2.65
Anguillarum	*Vibrio aestuarianus cardii*	15-061_5T2	*Vibrio aestuarianus* VN_10100	2.57	*Vibrio aestuarianus*VN-3828	2.67
Anguillarum	*Vibrio aestuarianus cardii*	15-064_3T2	*Vibrio aestuarianus* VN_10100	2.57	*Vibrio aestuarianus*VN-3828	2.68
Anguillarum	*Vibrio aestuarianus cardii*	15-064_4T2	*Vibrio aestuarianus* VN_10100	2.58	*Vibrio aestuarianus*VN-3828	2.67
Anguillarum	*Vibrio aestuarianus cardii*	15-075_1T2	*Vibrio aestuarianus*DSM 19606	2.32	*Vibrio aestuarianus*VN-2867	2.49
Anguillarum	*Vibrio aestuarianus cardii*	15-075_3T2	*Vibrio aestuarianus*VN_10100	2.56	*Vibrio aestuarianus*VN-3828	2.6
Anguillarum	*Vibrio aestuarianus francensis*	01-032	*Vibrio aestuarianus*DSM 19606	2.52	*Vibrio aestuarianus*VN-3828	2.59
Anguillarum	***Vibrio aestuarianus francensis***	**02-041T**	*Vibrio aestuarianus*VN_10100	2.61	*Vibrio aestuarianus*VN-3828	2.64
Anguillarum	*Vibrio aestuarianus francensis*	03-011-4B12	*Vibrio aestuarianus*VN_10100	2.54	*Vibrio aestuarianus*VN-3828	2.58
Anguillarum	*Vibrio aestuarianus francensis*	03-146-1B1	*Vibrio aestuarianus*DSM 19606	2.49	*Vibrio aestuarianus*VN-3828	2.55
Anguillarum	*Vibrio aestuarianus francensis*	07-084-3T1	*Vibrio aestuarianus*VN_10100	2.55	*Vibrio aestuarianus*VN-3828	2.6
Anguillarum	*Vibrio aestuarianus francensis*	07-139-1T1	*Vibrio aestuarianus*VN_10100	2.56	*Vibrio aestuarianus*VN-3828	2.59
Anguillarum	*Vibrio aestuarianus francensis*	08-081-5T1	*Vibrio aestuarianus*DSM 19606	2.48	*Vibrio aestuarianus*VN-3828	2.46
Anguillarum	*Vibrio aestuarianus francensis*	08-090-3T1	*Vibrio aestuarianus*DSM 19606	2.51	*Vibrio aestuarianus*VN-3828	2.65
Anguillarum	*Vibrio aestuarianus francensis*	08-093-3T1	*Vibrio aestuarianus*DSM 19606	2.56	*Vibrio aestuarianus*VN-2993	2.65
Anguillarum	*Vibrio aestuarianus francensis*	08-103-2T1	*Vibrio aestuarianus*VN_10100	2.58	*Vibrio aestuarianus*VN-3828	2.61
Anguillarum	*Vibrio aestuarianus francensis*	08-114-4T3	*Vibrio aestuarianus*DSM 19606	2.54	*Vibrio aestuarianus*VN-2993	2.61
Anguillarum	*Vibrio aestuarianus francensis*	08-127-5T1	*Vibrio aestuarianus*DSM 19606	2.53	*Vibrio aestuarianus*VN-3828	2.57
Anguillarum	*Vibrio aestuarianus francensis*	09-045-5T1	*Vibrio aestuarianus*VN_10100	2.51	*Vibrio aestuarianus*VN-3828	2.56

Anguillarum	*Vibrio aestuarianus francensis*	09-048-4T1	*Vibrio aestuarianus* DSM 19606	2.51	*Vibrio aestuarianus* VN-3828	2.59
Anguillarum	*Vibrio aestuarianus francensis*	10-075-3T2	*Vibrio aestuarianus* DSM 19606	2.5	*Vibrio aestuarianus* VN-3828	2.53
Anguillarum	*Vibrio aestuarianus francensis*	11-034-4T1	*Vibrio aestuarianus* VN_10100	2.53	*Vibrio aestuarianus* VN-3828	2.57
Anguillarum	*Vibrio aestuarianus francensis*	11-051-11T1	*Vibrio aestuarianus* VN_10100	2.53	*Vibrio aestuarianus* VN-3828	2.64
Anguillarum	*Vibrio aestuarianus francensis*	11-081-4MT2	*Vibrio aestuarianus* VN_10100	2.45	*Vibrio aestuarianus* VN-2993	2.47
Anguillarum	*Vibrio aestuarianus francensis*	11-183-10T2	*Vibrio aestuarianus* VN_10100	2.43	*Vibrio aestuarianus* VN-2993	2.48
Anguillarum	*Vibrio aestuarianus francensis*	12-128-1T3	*Vibrio aestuarianus* DSM 19606	2.55	*Vibrio aestuarianus* VN-3828	2.68
Anguillarum	*Vibrio aestuarianus francensis*	12-146-3T1	*Vibrio aestuarianus* VN_10100	2.56	*Vibrio aestuarianus* VN-3828	2.69
Anguillarum	*Vibrio aestuarianus francensis*	12-147-1T1	*Vibrio aestuarianus* DSM 19606	2.51	*Vibrio aestuarianus* VN-3828	2.48
Coralliilyticus	*V. coralliilyticus*	06-209	*Vibrio pectenicida* A365 EGS	1.8	*Vibrio corallilyticus* VN-3810	2.13
Coralliilyticus	*V. coralliilyticus*	06-210	*Vibrio pectenicida* A365 EGS	1.74	*Vibrio corallilyticus* VN-3810	2.21
Coralliilyticus	*V. coralliilyticus*	06-211	*Vibrio pectenicida* A365 EGS	1.61	*Vibrio corallilyticus* VN-3810	2.2
Coralliilyticus	*V. coralliilyticus*	06-221	*Vibrio neptunius* DSM 17183T	1.75	*Vibrio corallilyticus* VN-3810	2.19
Coralliilyticus	*V. coralliilyticus*	06-225	*Vibrio pectenicida* A365 EGS	1.74	*Vibrio corallilyticus* VN-3810	2.16
Coralliilyticus	*V. coralliilyticus*	**DSM 19607T**	*Vibrio pectenicida* A365 EGS	1.74	*Vibrio corallilyticus* VN-3810	2.14
Coralliilyticus	*V. coralliilyticus*	09-123_1T5	*Vibrio pectenicida* A365 EGS	1.66	*Vibrio corallilyticus* VN-3810	2.05
Coralliilyticus	*V. neptunius*	09-123_1T2	*Vibrio neptunius* DSM 17183T	2.03	*Vibrio corallilyticus* VN-3810	1.67
Coralliilyticus	*V. neptunius*	**DSM17183T**	*Vibrio neptunius* DSM 17183T	1.95	*Vibrio corallilyticus* VN-3810	1.81
Coralliilyticus	*V. ostreicida*	**CECT 7398T**	*Vibrio ostreicida* DSM 21434	2.56	*Vibrio xuii* VN-3825	1.5
Coralliilyticus	*V. ostreicida*	CECT 7399	*Vibrio ostreicida* DSM 21434	2.66	*Vibrio xuii* VN-3825	1.67
Harveyi	*V. alginolyticus*	**LMG 4409T**	*Vibrio alginolyticus* DSM 2171T	2.43	*Vibrio alginolyticus* VN-2769	2.6
Harveyi	*V. campbellii*	CIP 70.67	*Vibrio harveyi* VN_02926	2.37	*Vibrio harveyi* VN-2829	2.33
Harveyi	*V. harveyi*	04-018_1T1	*Vibrio harveyi* DSM 19623T	2.5	*Vibrio alginolyticus* VN-2658	2.22
Harveyi	*V. harveyi*	04-092_2T4	*Vibrio harveyi* DSM 19623T	2.51	*Vibrio alginolyticus* VN-2774	2.21
Harveyi	*V. harveyi*	04-107_1T1	*Vibrio harveyi* DSM 19623T	2.54	*Vibrio alginolyticus* VN-2774	2.21
Harveyi	*V. harveyi*	05-053_1T1	*Vibrio harveyi* DSM 19623T	2.53	*Vibrio alginolyticus* VN-2774	2.22
Harveyi	*V. harveyi*	05-074_1T3	*Vibrio harveyi* DSM 19623T	2.5	*Vibrio alginolyticus* VN-2774	2.29
Harveyi	*V. harveyi*	08-075_3T2	*Vibrio harveyi* DSM 19623T	2.55	*Vibrio harveyi* VN-2829	2.19
Harveyi	*V. harveyi*	08-077_4T2	*Vibrio harveyi* DSM 19623T	2.48	*Vibrio alginolyticus* VN-2654	2.14
Harveyi	*V. harveyi*	LMG7890	*Vibrio harveyi* DSM 19623T	2.45	*Vibrio alginolyticus* VN-2774	2.14
Harveyi	*V. jasicida*	CECT 8524	*Vibrio harveyi* VN_02926	2.54	*Vibrio harveyi* VN-2829	2.55
Harveyi	*V. jasicida*	07-078_1T2	*Vibrio harveyi* VN_02940	2.43	*Vibrio harveyi* VN-2829	2.44
Harveyi	*V. jasicida*	07-081_2T2	*Vibrio harveyi* VN_02926	2.27	*Vibrio harveyi* VN-2829	2.33
Harveyi	*V. jasicida*	07-109_T1	*Vibrio harveyi* VN_02940	2.52	*Vibrio harveyi* VN-2822	2.46
Harveyi	*V. mytili*	**DSM 19137T**	*Vibrio mytili* DSM 19137T	2.43	*Vibrio alginolyticus* VN-2685	2.16
Harveyi	*V. natriegens*	**CECT 526T**	*Vibrio natriegens* VN_02951	2.23	*Vibrio natriegens* VN-2951	2.43
Harveyi	*V. rotiferianus*	07-040_5T2	*Vibrio rotiferianus* DSM 17186T	2.26	*Vibrio harveyi* VN-2832	1.95

Harveyi	*V. rotiferianus*	07-103_1T3	*Vibrio rotiferianus* DSM 17186T	2.35	*Vibrio harveyi* VN-2832	2.15
Harveyi	*V. rotiferianus*	07-115_T1	*Vibrio rotiferianus* DSM 17186T	2.29	*Vibrio alginolyticus* VN-2775	2.1
Harveyi	*V. rotiferianus*	07-118_T1	*Vibrio rotiferianus* DSM 17186T	2.29	*Vibrio harveyi* VN-2829	2.17
Harveyi	*V. rotiferianus*	16-148_1T3	*Vibrio rotiferianus* DSM 17186T	2.26	*Vibrio harveyi* VN-2926	2.23
Harveyi	*V. rotiferianus*	7G5_1	*Vibrio rotiferianus* DSM 17186T	2.34	*Vibrio parahaemolyticus* VN-2679	2.18
Harveyi	*V. rotiferianus*	7H5_2	*Vibrio rotiferianus* DSM 17186T	2.34	*Vibrio parahaemolyticus* VN-2683	2.21
Harveyi	*V. rotiferianus*	8T1_2	*Vibrio rotiferianus* DSM 17186T	2.34	*Vibrio harveyi* VN-2829	2.26
Harveyi	*V. rotiferianus*	8T5_6	*Vibrio rotiferianus* DSM 17186T	2.33	*Vibrio harveyi* VN-2829	2.22
Harveyi	*V. rotiferianus*	8T6_4	*Vibrio rotiferianus* DSM 17186T	2.34	*Vibrio harveyi* VN-2829	2.19
Harveyi	*V. rotiferianus*	**DSM 17186T**	*Vibrio rotiferianus* DSM 17186T	2.3	*Vibrio parahaemolyticus* VN-2679	2.18
Mediterranei	*V. mediterranei*	DSM 13773	*Vibrio mediterranei* DSM 19502T	2.35	*Vibrio mediterranei* VN-3819	2.52
Orientalis	*V. brasiliensis*	**DSM 17184T**	*Vibrio brasiliensis* DSM 17184T	2.02	*Vibrio navarrensis* VN-3826	1.54
Orientalis	*V. orientalis*	**DSM 7897T**	*Vibrio orientalis* LMG 7897T	2.11	*Vibrio parahaemolyticus* VN-2826	1.63
Orientalis	*V. orientalis*	8F5_42	*Vibrio orientalis* LMG 7897T	2.04	*Vibrio alginolyticus* VN-2685	1.73
Orientalis	*V. orientalis*	8H6_11	*Vibrio orientalis* LMG7897T	2.03	*Vibrio alginolyticus* VN-2620	1.59
Orientalis	*V. orientalis*	8Z_12	*Vibrio orientalis* LMG 7897T	1.94	*Vibrio alginolyticus* VN-2685	1.79
Orientalis	*V. orientalis*	8Z_27	*Vibrio orientalis* LMG 7897T	1.96	*Vibrio alginolyticus* VN-2672	1.92

B.

			Bruker database		Erler database	
**Clade**	**Species**	**Strain**	**Organism best match**	**Log score**	**Organism best match**	**Log score**
-	***V. cortegadensis***	**DSM 102805T**	*Citrobacter freundii* 13158_2 CHB	1.41	*Vibrio parahaemolyticus* VN-3852	1.28
-	***V. tapetis tapetis***	**CECT 4600T**	*Lactobacillus crispatus* DSM 20584T	1.36	*Vibrio alginolyticus* VN-2784	1.37
-	*V. tapetis tapetis*	11.2	*Atopobium minutum* DSM 20586T	1.2	*Vibrio vulnificus* VN-3437	1.28
-	*V. tapetis tapetis*	2.3	*Ochrobactrum anthropi* DSM 20150	1.17	*Vibrio alginolyticus* VN-2559	1.21
-	*V. tapetis tapetis*	IS1	*Flavobacterium flevense* DSM 1076T	1.27	*Vibrio vulnificus* VN-3932	1.49
-	*V. tapetis tapetis*	IS5	*Photorhabdus temperata* DSM 14550T	1.3	*Vibrio navarrensis* VN-0256	1.45
-	*V. tapetis tapetis*	IS7	*Jonesia denitrificans* DSM 20603T	1.28	*Vibrio navarrensis* VN-3139	1.26
-	*V. tapetis tapetis*	IS8	*Elizabethkingia miricola* DSM 14571T	1.31	*Vibrio alginolyticus* VN-2784	1.36
-	*V. tapetis tapetis*	IS9	*Listeria innocua* CCUG 24928	1.23	*Vibrio navarrensis* VN-3139	1.31
-	*V. tapetis tapetis*	LP2	*Vibrio ostreicida* DSM 21433T	1.33	*Vibrio vulnificus* VN-3367	1.51
-	*V. tapetis tapetis*	P16b	*Photorhabdus temperata* DSM 14550T	1.21	*Vibrio vulnificus* VN-3446	1.21
-	*V. tapetis tapetis*	RP 1703	*Lactobacillus parabuchneri* DSM 5708	1.24	*Vibrio vulnificus* VN-3425	1.34
-	*V. tapetis tapetis*	RP 6301	*Aeromonas veronii* CECT 4257T	1.27	*Vibrio vulnificus* VN-3437	1.42
-	*V. tapetis tapetis*	UK6	*Photorhabdus temperata* DSM 14550T	1.26	*Vibrio vulnificus* VN-3398	1.36

Harveyi	*V. owensii*	**DSM 23055T**	*Vibrio harveyi* VN_02926	2.29	*Vibrio harveyi* VN-3801	2.5
Harveyi	*V. sagamiensis*	**DSM 25651T**	*Vibrio harveyi* VN_02940	1.81	*Vibrio harveyi* VN-2824	1.8
Harveyi	*V. sinaloensis*	**CECT 7298T**	*Vibrio mytili* DSM 19137T	1.72	*Vibrio alginolyticus* VN-2664	1.8
Mediterranei	*V. maritimus*	**DSM 26148T**	*Vibrio shilonii* DSM 13774T	1.88	*Vibrio mediterranei* VN-3819	2
Mediterranei	*V. variabilis*	**DSM 26147T**	*Vibrio shilonii* DSM 13774T	1.81	*Vibrio mediterranei* VN-3819	2
Orientalis	*V. bivalvicida*	14-105_2T1	*Vibrio brasiliensis* DSM 17184T	1.52	*Vibrio alginolyticus* VN-2736	1.39
Orientalis	*V. bivalvicida*	14-105_3T1	*Vibrio pectenicida* A365 EGS	1.58	*Vibrio alginolyticus* VN-2756	1.41
Orientalis	*V. bivalvicida*	14-106_1T1	*Vibrio brasiliensis* DSM 17184T	1.64	*Vibrio parahaemolyticus* VN-0053	1.51
Orientalis	*V. bivalvicida*	14-106_49T1	*Vibrio pectenicida* A365 EGS	1.59	*Vibrio alginolyticus* VN-2804	1.57
Orientalis	*V. bivalvicida*	**CECT 8855T**	*Vibrio brasiliensis* DSM 17184T	1.81	*Vibrio navarrensis* VN-3125	1.45
Orientalis	*V. bivalvicida*	CECT 8856	*Vibrio orientalis* LMG 7897T	1.71	*Vibrio alginolyticus* VN-2806	1.58
Orientalis	*V. europaeus*	07-038_2T2	*Vibrio brasiliensis* DSM 17184T	1.72	*Vibrio corallilyticus* VN-3810	1.52
Orientalis	*V. europaeus*	07-108_T1	*Vibrio brasiliensis* DSM 17184T	1.73	*Vibrio alginolyticus* VN-2637	1.55
Orientalis	*V. europaeus*	07-110_T1	*Vibrio brasiliensis* DSM 17184T	1.78	*Vibrio navarrensis* VN-3139	1.53
Orientalis	*V. europaeus*	07-112_T1	*Vibrio brasiliensis* DSM 17184T	1.74	*Vibrio corallilyticus* VN-3810	1.48
Orientalis	*V. europaeus*	07-115_T2	*Vibrio brasiliensis* DSM 17184T	1.61	*Vibrio alginolyticus* VN-2896	1.54
Orientalis	*V. europaeus*	07-116_T1	*Vibrio pectenicida* A365 EGS	1.58	*Vibrio fluvialis* VN-3830	1.55
Orientalis	*V. europaeus*	07-117_T1	*Vibrio brasiliensis* DSM 17184T	1.59	*Vibrio navarrensis* VN-3139	1.56
Orientalis	*V. europaeus*	07-119_T1	*Vibrio pectenicida* A365 EGS	1.72	*Vibrio fluvialis* VN-10028	1.51
Orientalis	*V. europaeus*	07-120_T1	*Vibrio brasiliensis* DSM 17184T	1.65	*Vibrio fluvialis* VN-10033	1.55
Orientalis	*V. europaeus*	07-121_1T1	*Vibrio brasiliensis* DSM 17184T	1.61	*Vibrio corallilyticus* VN-3810	1.54
Orientalis	*V. europaeus*	**CECT 8136T**	*Vibrio pectenicida* A365 EGS	1.72	*Vibrio fluvialis* VN-3173	1.58
Orientalis	*V. hepatarius*	**DSM 19134T**	*Vibrio orientalis* LMG 7897T	2.06	*Vibrio alginolyticus* VN-2685	1.73
Orientalis	*V. tubiashii*	**DSM 19142T**	*Vibrio brasiliensis* DSM 17184T	1.69	*Vibrio fluvialis* VN-3830	1.6

Incorrect identifications despite scores >2 are indicated by red text on a green background. Score values: 2.300–3.000 (Highly probable species-level identification); 2.000–2.229 (secure genus-level identification and a probable species-level identification); ≥ 1.700–1.999 (probable genus-level identification) and < 1.700 (unreliable identification).

Table S3.100 *Vibrio* strains used for the ~~MP VibrioBase~~EnviBase blind test.

Clade	Species	Strain number	Sampling date	Sampling location	Sample origin
-	*Alteromonas macleodi*	LMG 2843			
-	*V. cortegadensis*	DSM 102805	2005	Spain	Manila clam,*Ruditapesphilippinarum*
-	*V. galatheae*	LMG 28895T	2007	Solomon Islands	Mussel, surface
-	*V. tapetis tapetis*	RD0202	1992	Spain	European clam, *Ruditapes decussatus*
-	*V. tapetis tapetis*	RD0705	1994	Spain	European clam, *Ruditapes decussatus,*
Anguillarum	*V. aestuarianus*	LMG 7909	1983	USA	Pacific oyster, *Crassostrea gigas*
Anguillarum	*V. aestuarianus*	01/308	2001	France	Pacific oyster, *Crassostrea gigas*
Anguillarum	*V. aestuarianus*	LMG 24517	2002	France	Pacific oyster, *Crassostrea gigas*
Anguillarum	*V. aestuarianus*	03/058 1T1	2003	France	Pacific oyster *Crassostrea gigas*,
Anguillarum	*V. aestuarianus*	04/091 3T1	2004	France	Flat oyster, *Ostrea edulis*
Anguillarum	*V. aestuarianus*	07/115	2007	France	Pacific oyster, *Crassostrea gigas*
Anguillarum	*V. aestuarianus*	08/085 1T1	2008	France	Pacific oyster, *Crassostrea gigas*
Anguillarum	*V. aestuarianus*	08/162 1T1	2008	France	Pacific oyster, *Crassostrea gigas*
Anguillarum	*V. aestuarianus*	09/045 2T1	2009	France	Pacific oyster, *Crassostrea gigas*
Anguillarum	*V. aestuarianus*	10/075 9T2	2010	France	Pacific oyster, *Crassostrea gigas*
Anguillarum	*V. aestuarianus*	10/092 7MT1	2010	France	Pacific oyster, *Crassostrea gigas*
Anguillarum	*V. aestuarianus*	12/122 1T3	2012	France	Cockle, *Cerastoderma edule*
Anguillarum	*V. aestuarianus*	12/147 3T1	2012	France	Pacific oyster, *Crassostrea gigas*
Anguillarum	*V. aestuarianus*	15/061 4T4	2015	France	Cockles, *Cerastoderma edule*
Anguillarum	*V. aestuarianus*	18/033 5T1	2018	France	Cockles, *Cerastoderma edule*
Anguillarum	*V. aestuarianus*	18/047 1T2	2018	France	Pacific oyster, *Crassostrea gigas*
Anguillarum	*V. aestuarianus*	18/101 1T1	2018	France	Pacific oyster, *Crassostrea gigas*
Anguillarum	*V. anguillarum*	LMG 4437T	1956	Norway	Cod, *Gadus morhua*
Anguillarum	*V. salilacus*	JCM 19265	2013	China	Seawater
Coralliilyticus	*V. coralliilyticus*	LMG 20538	1998	Brazil	Scallop larvae, *Nodipecten nodosus*
Coralliilyticus	*V. coralliilyticus*	LMG 20548	1999	Brazil	Scallop larvae, *Nodipecten nodosus*
Coralliilyticus	*V. coralliilyticus*	LMG 21348	2001	Israel	Coral, *Pocillopora damicornis*
Coralliilyticus	*V. coralliilyticus*	DSM 19607T	1999	Tanzania	Coral, *Pocillopora damicornis*
Coralliilyticus	*V. coralliilyticus*	LMG 23696	2003	Australia	Coral, *Montipora sp.*
Coralliilyticus	*V. coralliilyticus*	06/209	2006	France	Pacific oyster, *Crassostrea gigas*
Coralliilyticus	*V. coralliilyticus*	09/123 1T5	2009	France	Pacific oyster, *Crassostrea gigas*
Coralliilyticus	*V. neptunius*	LMG 20613	1999	Belgium	Scallop larvae, *Nodipecten nodosus*
Coralliilyticus	*V. neptunius*	LMG 20614	1999	Belgium	Rotifer, *Brachionus plicatilis*

Coralliilyticus	*V. neptunius*	09/123 1T2	2009	France	Pacific oyster, *Crassostrea gigas*
Coralliilyticus	*V. neptunius*	DSM 17183T	1998	Brazil	Scallop larvae, *Nodipecten nodosus*
Coralliilyticus	*V. ostreicida*	CECT 7399T	1999	Spain	Mollusc hatchery
Coralliilyticus	*V. ostreicida*	CECT 7398	1999	Spain	Mollusc hatchery
Harveyi	*V. jasicida-like*	F5-C01	2015	France	Seawater
Harveyi	*V. alginolyticus*	LMG 4409T	Before 1973	Japan	Spoiled horse mackerel, *Trachurus trachurus*
Harveyi	*V. azureus*	LMG 25266	2006	Japan	Seawater
Harveyi	*V. campbellii*	CIP 70.67	1968	Japan	Seawater
Harveyi	*V. diabolicus*	14/079 2T1	2014	France	Abalone, *Haliotis tuberculata*
Harveyi	*V. harveyi*	ORM 4	1998	France	European clam, *Ruditapes decussatus*
Harveyi	*V. harveyi*	08/075 3T2	2008	France	Pacific oyster, *Crassostrea gigas*
Harveyi	*V. harveyi*	LMG 4044T	Before 1973	USA	Abalone, *Haliotis tuberculata*
Harveyi	*V. harveyi*	LMG 7890	1982	USA	Brown shark, *Carcharhinus plumbeus*
Harveyi	*V. harveyi*	F5-F11	2015	France	Seawater
Harveyi	*V. harveyi*	OA01	2015	France	Pacific oyster, *Crassostrea gigas*
Harveyi	*V. harveyi*	OD03	2015	France	Pacific oyster, *Crassostrea gigas*
Harveyi	*V. harveyi*	OG11	2015	France	Pacific oyster, *Crassostrea gigas*
Harveyi	*V. hyuagaensis*	LMG 28466	2010	Japan	Seawater
Harveyi	*V. jasicida*	LMG 25398	1999	New Zealand	Rock robster, *Jasus verreaux*
Harveyi	*V. jasicida*	CECT 8524	2004	Mexico	Spotted rose snapper, *Lutjanus guttatus*
Harveyi	*V. jasicida*	07/040 1T2	2007	France	Pacific oyster, *Crassostrea gigas*
Harveyi	*V. jasicida*	07/078 1T2	2007	France	Pacific oyster, *Crassostrea gigas*
Harveyi	*V. jasicida*	F1A12	2015	France	Seawater
Harveyi	*V. jasicida*	F1-CO8	2015	France	Seawater
Harveyi	*V. jasicida*	F5-G08	2015	France	Seawater
Harveyi	*V. jasicida*	F5-H11	2015	France	Seawater
Harveyi	*V. mytili*	DSM 19137T	Before 1985	Spain	Mussel, *Mytilus edulis*
Harveyi	*V. natriegens*	CECT 526T	1983	USA	Salt marsh mud
Harveyi	*V. owensii*	DSM 23055Y	2007	Australia	Spiny lobster larva, *Panulirus ornatus*
Harveyi	*V. owensii*	F1-D04	2015	France	Seawater
Harveyi	*V. owensii*	OA03	2015	France	Pacific oyster, *Crassostrea gigas*
Harveyi	*V. owensii*	ZG08	2015	France	Zooplankton
Harveyi	*V. rotiferianus*	DSM 17186T	2005	Belgium	Rotifer, *Brachionus plicatilis*
Harveyi	*V. rotiferianus*	16/148 1T3	2016	France	Pacific oyster, *Crassostrea gigas*
Harveyi	*V. rotiferianus*	7H5_2	2014	France	Pacific oyster, *Crassostrea gigas*
Harveyi	*V. rotiferianus*	8T1_2	2014	France	Pacific oyster, *Crassostrea gigas*
Harveyi	*V. rotiferianus*	OB06	2015	France	Pacific oyster, *Crassostrea gigas*
Harveyi	*V. rotiferianus*	OE12	2015	France	Pacific oyster, *Crassostrea gigas*

Harveyi	*V. rotiferianus*	OG05	2015	France	Pacific oyster, *Crassostrea gigas*
Harveyi	*V. rotiferianus*	OG10	2015	France	Pacific oyster, *Crassostrea gigas*
Harveyi	*V. rotiferianus*	OH08	2015	France	Pacific oyster, *Crassostrea gigas*
Harveyi	*V. sagamiensis*	DSM 25651T	2008	Pacific Ocean	Seawater,
Mediterranei	*V. barjei*	LMG 29358	2011	Spain	Abalone, *Haliotis tuberculata*
Mediterranei	*V. mediterranei*	DSM 13773	Before 1981	Spain	Sediment
Nereis	*V. nereis*	LMG 3895T	Before 1973	Hawaii	Seawater
Nereis	*V. xuii*	DSM 17185T	Before 2005	China	Shrimp culture water
Orientalis	*V. bivalvicida*	CECT 8855T	2008	Spain	European clam, *Ruditapes decussatus*
Orientalis	*V. bivalvicida*	14/105 3T2	2014	France	Manila clam,*Ruditapes philippinarum*
Orientalis	*V. bivalvicida*	14/106 1T1	2014	France	Cockle, *Cerastoderma edule*
Orientalis	*V. bivalvicida*	14/106 49T2	2014	France	Cockle, *Cerastoderma edule*
Orientalis	*V. brasiliensis*	DSM 17184T	2005	Brazil	Scallop larvae *Nodipecten nodosus*,
Orientalis	*V. europaeus*	CECT 8136	2001	Spain	Flat oyster, *Ostrea edulis*
Orientalis	*V. europaeus*	04/002 1T2	2004	France	Abalone, *Haliotis tuberculata*
Orientalis	*V. europaeus*	14/106 32T3	2014	France	Cockle, *Cerastoderma edule*
Orientalis	*V. europaeus*	14/106 37T1	2014	France	Cockle, *Cerastoderma edule*
Orientalis	*V. europaeus*	14/107 4T1	2014	France	Manila clam, *Ruditapes philippinarum*
Orientalis	*V. europaeus*	14/107 7T2	2014	France	Manila clam, *Ruditapes philippinarum*
Orientalis	*V. europaeus*	16/148 1T1	2016	France	Pacific oyster, *Crassostrea gigas*
Orientalis	*V. hepatarius*	DSM 19134	2007	Ecuador	Shrimp, *Litopenaeus vannamei*
Orientalis	*V. orientalis*	LMG 7897T	1984	Yellow Sea	Seawater
Orientalis	*V. sinaloensis*	CECT 7298T	Before 2003	Mexico	Spotted rose snapper, *Lutjanus guttatus*
Orientalis	*V. tubiashii*	DSM 19142	2007	France	Hard clam, *Mercenaria mercenaria*
Orientalis	*V. tubiashii*	14/106 35T1	2014	France	Cockle, *Cerastoderma edule*
Orientalis	*V. tubiashii*	14/107 6T2	2014	France	Manila clam, *Ruditapes philippinarum*
Orientalis	*V. tubiashii*	LMG 11229	Before 1991	USA	Eastern Oyster, *Crassostrea virginica*
Splendidus	*V. chagasii*	OF05	2015	France	Pacific oyster, *Crassostrea gigas*
Splendidus	*V. splendidus*	LMG 4042	before 1958	Unknown	Marine fish

Table S4. Best matching score values of the *Vibrio* strains present in the newly created databases used for MP the VibrioBase blind test (**A**) for species present in both or in at least one database, (**B**) for species absent from both databases**.**

**A.**			~~**VibrioBase LNR**~~**EnviBase**				**EnviBase** ~~**VibrioBase LNR**~~ **+** ~~**Biotyper**~~ **Bruker + Erler databases**			
**Species**	**Strain**	**Lab**	**Direct transfer**	**Score**	**Protein extraction**	**Score**	**Direct transfer**	**Score**	**Protein extraction**	**Score**
Vibrio aestuarianus	03-008T	1	*V. aestuarianus* 08-103 2T1	2.63	*V. aestuarianus* 16-055 5T1	2.64	*V. aestuarianus* 08-103 2T1	2.63	*V. aestuarianus* 16-055 5T1	2.64
		2	*V. aestuarianus* 16-055 5T1	2.52	*V. aestuarianus* 03-008 T	2.44	*V. aestuarianus* 16-055 5T1	2.52	*V. aestuarianus* 03-008 T	2.44
		3	*V. aestuarianus* 16-055 5T1	2.6	*V. aestuarianus* 16-055 5T1	2.66	*V. aestuarianus* 16-055 5T1	2.6	*V. aestuarianus* 16-055 5T1	2.66
	01/308	1	*V. aestuarianus* 10-075 3T2	2.6	*V. aestuarianus* 08-093 3T1	2.69	*V. aestuarianus* 10-075 3T2	2.6	*V. aestuarianus* 08-093 3T1	2.69
		2	*V. aestuarianus* 08-103 2T1	2.57	*V. aestuarianus* 08-093 3T1	2.52	*V. aestuarianus* 08-103 2T1	2.57	*V. aestuarianus 08-093 3T1*	2.52
		3	*V. aestuarianus* 10-075 3T2	2.34	*V. aestuarianus* 09-048 4T1	2.5	*V. aestuarianus* 10-075 3T2	2.34	*V. aestuarianus* 09-048 4T1	2.5
	02-041	1	*V. aestuarianus* 08-103 2T1	2.65	*V. aestuarianus* 01-032	2.66	*V. aestuarianus* 08-103 2T1	2.65	*V. aestuarianus* 01-032	2.66
		2	*V. aestuarianus* 07-084 3T1	2.39	*V. aestuarianus* 12-146 3T1	2.54	*V. aestuarianus* 07-084 3T1	2.39	*V. aestuarianus* 12-146 3T1	2.54
		3	*V. aestuarianus* 01-032	2.43	*V. aestuarianus* 01-032	2.63	*V. aestuarianus* 01-032	2.43	*V. aestuarianus* 01-032	2.63
	03-058_1T1	1	*V. aestuarianus* 08-103 2T1	2.66	*V. aestuarianus* 10-075 3T2	2.53	*V. aestuarianus* 08-103 2T1	2.66	*V. aestuarianus* 10-075 3T2	2.53
		2	*V. aestuarianus* 09-045 5T1	2.5	*V. aestuarianus* 08-103 2T1	2.69	*V. aestuarianus* 09-045 5T1	2.5	*V. aestuarianus* 08-103 2T1	2.69
		3	*V. aestuarianus* 09-045 5T1	2.37	*V. aestuarianus* 11-034 4T1	2.7	*V. aestuarianus* 09-045 5T1	2.37	*V. aestuarianus* 11-034 4T1	2.7
	04-091 3T1	1	*V. aestuarianus* 08-103 2T1	2.61	*V. aestuarianus* 11-081 4MT2	2.49	*V. aestuarianus* 08-103 2T1	2.61	*V. aestuarianus* 11-081 4MT2	2.49
		2	*V. aestuarianus* 08-103 2T1	2.53	*V. aestuarianus* 07-084 3T1	2.58	*V. aestuarianus* 08-103 2T1	2.53	*V. aestuarianus* 07-084 3T1	2.58
		3	*V. aestuarianus* 09-045 5T1	2.54	*V. aestuarianus* 02-041	2.6	*V. aestuarianus* 09-045 5T1	2.54	*V. aestuarianus* 02-041	2.6
	07-115	1	*V. aestuarianus* 08-103 2T1	2.58	*V. aestuarianus* 11-034 4T1	2.6	*V. aestuarianus* 08-103 2T1	2.58	*V. aestuarianus* 11-034 4T1	2.6
		2	*V. aestuarianus* 09-045 5T1	2.45	*V. aestuarianus* 12-146 3T1	2.56	*V. aestuarianus* 09-045 5T1	2.45	*V. aestuarianus* 12-146 3T1	2.56
		3	*V. aestuarianus* 02-041	2.6	*V. aestuarianus* 08-103 2T1	2.61	*V. aestuarianus* 02-041	2.6	*V. aestuarianus* 08-103 2T1	2.61
	08-085_1T1	1	*V. aestuarianus* 15-061 5T2	2.64	*V. aestuarianus* 08-081 5T1	2.36	*V. aestuarianus* 15-061 5T2	2.64	*V. aestuarianus* 08-081 5T1	2.36
		2	*V. aestuarianus* 10-075 3T2	2.46	*V. aestuarianus* 10-075 3T2	2.52	*V. aestuarianus* 10-075 3T2	2.46	*V. aestuarianus* 10-075 3T2	2.52
		3	*V. aestuarianus* 10-075 3T2	2.49	*V. aestuarianus* 16-055 5T1	2.64	*V. aestuarianus* 10-075 3T2	2.49	*V. aestuarianus* 16-055 5T1	2.64
	08-162 1T1	1	*V. aestuarianus* 08-103 2T1	2.66	*V. aestuarianus* 08-127 5T1	2.45	*V. aestuarianus* 08-103 2T1	2.66	*V. aestuarianus* 08-127 5T1	2.45
		2	*V. aestuarianus* 01-032	2.53	*V. aestuarianus* 12-146 3T1	2.66	*V. aestuarianus* 01-032	2.53	*V. aestuarianus* 12-146 3T1	2.66
		3	*V. aestuarianus* 15-064 4T2	2.51	*V. aestuarianus* 12-146 3T1	2.67	*V. aestuarianus* 15-064 4T2	2.51	*V. aestuarianus* 12-146 3T1	2.67
	09-045 2T1	1	*V. aestuarianus* 08-103 2T1	2.66	*V. aestuarianus* 08-127 5T1	2.45	*V. aestuarianus* 08-103 2T1	2.66	*V. aestuarianus* 08-127 5T1	2.45
		2	*V. aestuarianus* 12-147 1T1	2.44	*V. aestuarianus* 03-011 4B12	2.66	*V. aestuarianus* 12-147 1T1	2.44	*V. aestuarianus* 03-011 4B12	2.66
		3	*V. aestuarianus* 11-081 4MT2	2.31	*V. aestuarianus* 12-146 3T1	2.63	*V. aestuarianus* 11-081 4MT2	2.31	*V. aestuarianus* 12-146 3T1	2.63
	10-075 9T2	1	*V. aestuarianus* 08-093 3T1	2.58	*V. aestuarianus* 08-127 5T1	2.54	*V. aestuarianus* 08-093 3T1	2.58	*V. aestuarianus* 08-127 5T1	2.54
		2	*V. aestuarianus* 08-103 2T1	2.57	*V. aestuarianus* 08-090 3T1	2.61	*V. aestuarianus* 08-103 2T1	2.57	*V. aestuarianus* 08-090 3T1	2.61
		3	*V. aestuarianus* 15-064 4T2	2.5	*V. aestuarianus* 02-041	2.52	*V. aestuarianus* 15-064 4T2	2.5	*V. aestuarianus* 02-041	2.52
	10-092 7MT1	1	*V. aestuarianus* 16-055 5T1	2.5	*V. aestuarianus 08-127 5T1*	2.56	*V. aestuarianus* 16-055 5T1	2.5	*V. aestuarianus* 08-127 5T1	2.56

		2	*V. aestuarianus* 10-075 3T2/ *V. aestuarianus* 12-122 3T3	2.51	*V. aestuarianus* 11-034 4T1	2.62	*V. aestuarianus* 10-075 3T2/ *V. aestuarianus* 12-122 3T3	2.51	*V. aestuarianus* 11-034 4T1	2.62
		3	*V. aestuarianus* 16-055 5T1	2.47	*V. aestuarianus* 08-090 3T1	2.57	*V. aestuarianus*16-055 5T1	2.47	*V. aestuarianus* 08-090 3T1	2.57
		1	*V. aestuarianus* 15-061 5T2	2.59	*V. aestuarianus* 09-048 4T1	2.58	*V. aestuarianus* 15-061 5T2	2.59	*V. aestuarianus* 09-048 4T1	2.58
	12-122_1T3	2	*V. aestuarianus* 12-122 1T1	2.38	*V. aestuarianus* 03-008 T	2.53	*V. aestuarianus* 12-122 1T1	2.38	*V. aestuarianus* 03-008 T	2.53
		3	*V. aestuarianus* 15-064 4T2/*V. aestuarianus* 02-041	2.07	*V. aestuarianus* 15-061 1T1	2.66	*V. aestuarianus* 15-064 4T2/ *V. aestuarianus* 02-041	2.07	*V. aestuarianus* 15-061 1T1	2.66
		1	*V. aestuarianus* 08-103 2T1	2.64	*V. aestuarianus* 11-051 11T1	2.34	*V. aestuarianus* 08-103 2T1	2.64	*V. aestuarianus* 11-051 11T1	2.34
	12-147 3T1	2	*V. aestuarianus* 10-075 3T2	2.36	*V. aestuarianus* 12-146 3T1	2.66	*V. aestuarianus* 10-075 3T2	2.36	*V. aestuarianus* 12-146 3T1	2.66
		3	*V. aestuarianus* 12-146 3T1	2.59	*V. aestuarianus* 12-146 3T1	2.64	*V. aestuarianus* 12-146 3T1	2.59	*V. aestuarianus* 12-146 3T1	2.64
		1	*V. aestuarianus* 15-061 1T1	2.62	*V. aestuarianus* 12-122 3T3	2.6	*V. aestuarianus* 15-061 1T1	2.62	*V. aestuarianus* 12-122 3T3	2.6
	15-061 4T4	2	*V. aestuarianus*03-008 T	2.45	*V. aestuarianus* 15-061 1T1	2.63	*V. aestuarianus* 03-008 T	2.45	*V. aestuarianus* 15-061 1T1	2.63
		3	*V. aestuarianus* 15-061 5T2	2.51	*V. aestuarianus* 15-061 1T1	2.64	*V. aestuarianus* 15-061 5T2	2.51	*V. aestuarianus* 15-061 1T1	2.64
		1	*V. aestuarianus* 15-075 1T2	2.41	*V. aestuarianus* 15-075 1T2	2.35	*V. aestuarianus* 15-075 1T2	2.41	*V. aestuarianus* 15-075 1T2	2.35
	18-033 5T1	2	*V. aestuarianus* 15-075 1T2	2.53	*V. aestuarianus* 15-075 1T2	2.52	*V. aestuarianus* 15-075 1T2	2.53	*V. aestuarianus* 15-075 1T2	2.52
		3	*V. aestuarianus* 15-075 1T2	2.09	*V. aestuarianus* 15-075 1T2	2.43	*V. aestuarianus* 15-075 1T2	2.09	*V. aestuarianus* 15-075 1T2	2.43
		1	*V. aestuarianus* 11-081 4MT2	2.43	*V. aestuarianus* 11-081 4MT2	2.28	*V. aestuarianus* 11-081 4MT2	2.43	*V. aestuarianus* 11-081 4MT2	2.28
	18-047_1T2	2	*V. aestuarianus* 12-147 1T1	2.45	*V. aestuarianus* 12-146 3T1	2.44	*V. aestuarianus* 12-147 1T1	2.45	*V. aestuarianus* 12-146 3T1	2.44
		3	*V. aestuarianus* 12-128 1T3	2.35	*V. aestuarianus* 16-055 5T1	2.62	*V. aestuarianus* 12-128 1T3	2.35	*V. aestuarianus* 16-055 5T1	2.62
		1	*V. aestuarianus* 15-061 5T2	2.6	*V. aestuarianus* 08-127 5T1	2.55	*V. aestuarianus* 15-061 5T2	2.6	*V. aestuarianus* 08-127 5T1	2.55
	18-101 1T1	2	*V. aestuarianus* 02-041	2.56	*V. aestuarianus* 16-055 5T1	2.46	*V. aestuarianus* 02-041	2.56	*V. aestuarianus* 16-055 5T1	2.46
		3	*V. aestuarianus* 15-061 4T2	2.41	*V. aestuarianus* 10-075 3T2	2.42	*V. aestuarianus* 15-061 4T2	2.41	*V. aestuarianus* 10-075 3T2	2.42
		1	*V. coralliilyticus* 06-225	2.43	*V. coralliilyticus* 06-211	2.27	*V. coralliilyticus* 06-225	2.43	*V. coralliilyticus* 06-211	2.27
	LMG20538	2	*V. coralliilyticus* 06-225	2.5	*V. coralliilyticus* 06-225	2.5	*V. coralliilyticus* 06-225	2.5	*V. coralliilyticus* 06-225	2.5
		3	*V. coralliilyticus* 06-225	2.29	*V. coralliilyticus* 06-225	2.39	*V. coralliilyticus* 06-225	2.29	*V. coralliilyticus* 06-225	2.39
		1	*V. coralliilyticus* 06-225	2.41	*V. coralliilyticus* 06-211	2.41	*V. coralliilyticus* 06-225	2.41	*V. coralliilyticus* 06-211	2.41
	LMG20548	2	*V. coralliilyticus* LMG19607	2.14	*V. coralliilyticus* 06-225	2.51	*V. coralliilyticus* LMG19607	2.14	*V. coralliilyticus* 06-225	2.51
		3	*V. coralliilyticus* 06-209	2.41	*V. coralliilyticus* LMG19607T	2.47	*V. coralliilyticus* 06-209	2.41	*V. coralliilyticus* LMG19607T	2.47
		1	*V. coralliilyticus* 06-211	2.15	*V. coralliilyticus* 06-211	2.26	*V. coralliilyticus* 06-211	2.15	*V. coralliilyticus* 06-211	2.26
V. coralliilyticus	LMG21348	2	*V. coralliilyticus* 06-211	2.33	*V. coralliilyticus* 06-225	2.38	*V. coralliilyticus* 06-211	2.33	*V. coralliilyticus* 06-225	2.38
		3	*V. coralliilyticus* 09-123 1T5	2.24	*V. coralliilyticus* 06-211	2.44	*V. coralliilyticus* 09-123 1T5	2.24	*V. coralliilyticus* 06-211	2.44
		1	*V. coralliilyticus* 06-211	2.05	*V. coralliilyticus* 06-225	2.31	*V. coralliilyticus* 06-211	2.05	*V. coralliilyticus* 06-225	2.31
	DSM19607T	2	*V. coralliilyticus* 06-211	2.42	*V. coralliilyticus* 06-211	2.43	*V. coralliilyticus* 06-211	2.42	*V. coralliilyticus* 06-211	2.43
		3	*V. coralliilyticus* LMG19607	2.00	*V. coralliilyticus* 06-225	2.39	*V. coralliilyticus* LMG19607	2.00	*V. coralliilyticus* 06-225	2.39
		1	*V. coralliilyticus* 06-225	2.41	*V. coralliilyticus* 06-211	2.2	*V. coralliilyticus* 06-225	2.41	*V. coralliilyticus* 06-211	2.2
	06-209	2	*V. coralliilyticus* 06-211	2.41	*V. coralliilyticus* 06-209	2.26	*V. coralliilyticus* 06-211	2.41	*V. coralliilyticus* 06-209	2.26
		3	*V. coralliilyticus* 06-225_66	2.28	*V. coralliilyticus* 06-210	2.5	*V. coralliilyticus* 06-225	2.28	*V. coralliilyticus* 06-210	2.5

	LMG23696	1	*V. coralliilyticus* 06-225	2.38	*V. coralliilyticus* 06-225	2.32	*V. coralliilyticus* 06-225	2.38	*V. coralliilyticus* 06-225	2.32
		2	*V. coralliilyticus* 06-211	2.41	*V. coralliilyticus* 06-225	2.43	*V. coralliilyticus* 06-211	2.41	*V. coralliilyticus* 06-225	2.43
		3	*V. coralliilyticus* 06-225	2.28	*V. coralliilyticus* LMG19607T	2.4	*V. coralliilyticus* 06-225	2.28	*V. coralliilyticus* LMG19607T	2.4
	09-123_1T5	1	*V. coralliilyticus* 06-211	2.42	*V. coralliilyticus* 06-225	1.71	*V. coralliilyticus* 06-211	2.42	*V. coralliilyticus* 06-225	1.71
		2	*V. coralliilyticus* 06-211	2.3	*V. coralliilyticus* 06-225	2.5	*V. coralliilyticus* 06-211	2.3	*V. coralliilyticus* 06-225	2.5
		3	*V. coralliilyticus* 06-225	2.03	*V. coralliilyticus* 06-211	2.21	*V. coralliilyticus* 06-225	2.03	*V. coralliilyticus* 06-211	2.21
V. neptunius	LMG20613	1	*V. neptunius* 09-123 1T2	2.47	*V. neptunius* 09-123 1T2	2.44	*V. neptunius* 09-123 1T2	2.47	*V. neptunius* 09-123 1T2	2.44
		2	*V. neptunius* 09-123 1T2	2.25	*V. neptunius* 09-123 1T2	2.52	*V. neptunius* 09-123 1T2	2.25	*V. neptunius* 09-123 1T2	2.52
		3	*V. neptunius* 09-123 1T2	2.16	*V. neptunius* DSM17183	2.32	*V. neptunius* 09-123 1T2	2.16	*V. neptunius DSM17183*	2.32
	LMG20614	1	*V. neptunius* 09-123 1T2	2.35	*V. neptunius* 09-123 1T2	2.39	*V. neptunius* 09-123 1T2	2.35	*V. neptunius* 09-123 1T2	2.39
		2	*V. neptunius* 09-123 1T2	2.31	*V. neptunius* 09-123 1T2	2.43	*V. neptunius* 09-123 1T2	2.31	*V. neptunius* 09-123 1T2	2.43
		3	*V. neptunius* DSM17183	2.04	*V. neptunius* DSM17183	2.38	*V. neptunius* DSM17183	2.04	*V. neptunius* DSM17183	2.38
	09-123 1T2	1	*V. neptunius* 09-123 1T2	2.36	*V. neptunius* DSM17183	2.44	*V. neptunius* 09-123 1T2	2.36	*V. neptunius* DSM17183	2.44
		2	*V. neptunius* 09-123 1T2	2.34	*V. neptunius* 09-123 1T2	2.27	*V. neptunius* 09-123 1T2	2.34	*V. neptunius* 09-123 1T2	2.27
		3	*V. neptunius* DSM17183	2.3	*V. neptunius* DSM17183	2.15	*V. neptunius* DSM17183	2.3	*V. neptunius* DSM17183	2.15
	DSM17183	1	*V. neptunius* 09-123 1T2	2.31	*V. neptunius* DSM17183	1.9	*V. neptunius* 09-123 1T2	2.31	*V. neptunius* DSM17183	1.9
		2	*V. neptunius* 09-123 1T2	2.35	*V. neptunius* 09-123 1T2	2.46	*V. neptunius* 09-123 1T2	2.35	*V. neptunius* 09-123 1T2	2.46
		3	*V. neptunius* DSM17183	2.09	*V. neptunius* DSM17183	2.49	*V. neptunius* DSM17183	2.09	*V. neptunius* DSM17183	2.49
V. ostreicida	CECT7398	1	*V. ostreicida* CECT7398	2.13	*V. ostreicida* CECT7398	2.10	*V. ostreicida* CECT7398	2.13	*V. ostreicida* CECT7398	2.10
		2	*V. ostreicida* CECT7398	2.14	*V. ostreicida* CECT7399	2.46	*V. ostreicida* DSM21434T[a]	2.32	*V. ostreicida* CECT7399	2.46
		3	*V. ostreicida* CECT7398	2.03	*V. ostreicida* CECT7398	2.32	*V. ostreicida* CECT7398	2.03	*V. ostreicida* DSM21434T[a]	2.35
	CECT7399	1	*V. ostreicida* CECT7398	2.51	*V. ostreicida* CECT7398	2.35	*V. ostreicida* CECT7398	2.51	*V. ostreicida* CECT7398	2.35
		2	*V. ostreicida* CECT7399	2.45	*V. ostreicida* CECT7399	2.32	*V. ostreicida* DSM21433T[a]	2.52	*V. ostreicida* CECT7399	2.32
		3	*V. ostreicida* CECT7399	2.02	*V. ostreicida* CECT7398	2.37	*V. ostreicida* CECT7399	2.02	*V. ostreicida* CECT7398	2.37
V. alginolyticus	LMG4409T	1	*V. alginolyticus* LMG4409	2.56	*V. alginolyticus* LMG4409	2.55	*V. alginolyticus* LMG4409	2.56	*V. alginolyticus* LMG4409	2.55
		2	*V. alginolyticus* LMG4409	2.24	*V. alginolyticus* LMG4409	2.34	*V. alginolyticus* VN2748[b]	2.44	*V. alginolyticus* VN2562 [b]	2.42
		3	*V. alginolyticus* LMG4409	2.34	*V. alginolyticus* LMG4409	2.37	*V. alginolyticus* LMG4409	2.34	*V. alginolyticus* LMG4409	2.37
V. campbellii	CIP70.67	1	*V. campbellii* CIP70.67	2.14	*V. campbellii* CIP70.67	2.5	*V. campbellii* CIP70.67	2.14	*V. campbellii* CIP70.67	2.5
		2	*V. campbellii* CIP70.67	2.12	*V. campbellii* CIP70.67	2.38	*V. campbellii* CIP70.67	2.12	*V. campbellii* CIP70.67	2.38
		3	*V. campbellii* CIP70.67	2.35	*V. campbellii* CIP70.67	2.36	*V. campbellii* CIP70.67	2.35	*V. campbellii* CIP70.67	2.36
V. harveyi	ORM 4	1	*V. harveyi* 05-074 1T3	2.56	*V. harveyi* 04-018 1T1	2.63	*V. harveyi* 05-074 1T3	2.56	*V. harveyi* 04-018 1T1	2.63
		2	*V. harveyi* 04-107 1T1	2.19	*V. harveyi* 05-053 1T1	2.39	*V. harveyi* 04-107 1T1	2.19	*V. harveyi* 05-053 1T1	2.39
		3	*V. harveyi* 04-107 1T1	2.47	*V. harveyi* 04-107 1T1	2.66	*V. harveyi* 04-107 1T1	2.47	*V. harveyi* 04-107 1T1	2.66
	08-075_3T2	1	*V. harveyi* 04-092 2T4	2.44	*V. harveyi* 08-075 3T2	2.59	*V. harveyi* 04-092 2T4	2.44	*V. harveyi* 08-075 3T2	2.59
		2	*V. harveyi* 04-107 1T1	2.36	*V. harveyi* 05-074 1T3	2.43	*V. harveyi* 04-107 1T1	2.36	*V. harveyi* 05-074 1T3	2.43
		3	*V. harveyi* 08-075 3T2	2.41	*V. harveyi* 08-075 3T2	2.44	*V. harveyi* 08-075 3T2	2.41	*V. harveyi* 08-075 3T2	2.44
	LMG4044	1	*V. harveyi* 04-092 2T4	2.49	*V. harveyi* 05-053 1T1	2.56	*V. harveyi* 04-092 2T4	2.49	*V. harveyi* 05-053 1T1	2.56
		2	*V. harveyi 04-107 1T1*	2.41	*V. harveyi 05-074 1T3*	2.33	*V. harveyi 04-107 1T1*	2.41	*V. harveyi 05-074 1T3*	2.33

		3	*V. harveyi 08-077 4T2*	2.24	*V. harveyi 05-053 1T1*	2.44	*V. harveyi 08-077 4T2*	2.24	*V. harveyi 05-053 1T1*	2.44
	LMG7890	1	*V. harveyi LMG7890*	2.41	*V. harveyi LMG7890*	2.57	*V. harveyi LMG7890*	2.41	*V. harveyi LMG7890*	2.57
		2	*V. harveyi LMG7890*	2.01	*V. harveyi LMG7890*	2.33	*V. harveyi LMG7890*	2.01	*V. harveyi LMG7890*	2.33
		3	*V. harveyi 05-074 1T3*	1.98	*V. harveyi LMG7890*	2.57	*V. harveyi 05-074 1T3*	1.98	*V. harveyi LMG7890*	2.57
	F5-F11	1	*V. harveyi 04-092 2T4*	2.37	*V. harveyi 08-075 3T2*	2.49	*V. harveyi 04-092 2T4*	2.37	*V. harveyi 08-075 3T2*	2.49
		2	*V. harveyi LMG7890*	2.07	*V. harveyi 05-074 1T3*	2.44	*V. harveyi LMG7890*	2.07	*V. harveyi 05-074 1T3*	2.44
		3	*V. harveyi LMG7890*	2.38	*V. harveyi 05-074 1T3*	2.25	*V. harveyi LMG7890*	2.38	*V. harveyi 05-074 1T3*	2.25
	OA01	1	*V. harveyi 08-077 4T2*	2.31	*V. harveyi 08-077 4T2*	2.51	*V. harveyi 08-077 4T2*	2.31	*V. harveyi 08-077 4T2*	2.51
		2	*V. harveyi 04-107 1T1*	2.37	*V. harveyi 08-077 4T2*	2.33	*V. harveyi 04-107 1T1*	2.37	*V. harveyi 08-077 4T2*	2.33
		3	*V. harveyi 04-107 1T1*	2.47	*V. harveyi 04-107 1T1*	2.53	*V. harveyi 04-107 1T1*	2.47	*V.harveyi 04-107 1T1*	2.53
	OD03	1	*V. harveyi 05-074 1T3*	2.44	*V. harveyi LMG7890*	2.54	*V. harveyi 05-074 1T3*	2.44	*V. harveyi LMG7890*	2.54
		2	*V. harveyi 08-077 4T2*	2.21	*V. harveyi 08-077 4T2*	2.38	*V. harveyi 08-077 4T2*	2.21	*V. harveyi 08-077 4T2*	2.38
		3	*V. harveyi 08-077 4T2*	2.41	*V. harveyi 08-077 4T2*	2.33	*V. harveyi 08-077 4T2*	2.41	*V. harveyi 08-077 4T2*	2.33
	OG11	1	*V. harveyi 08-077 4T2/ V. harveyi 04-107 1T1*	2.47	*V. harveyi 08-075 3T2*	2.57	*V. harveyi 08-077 4T2/ V. harveyi 04-107 1T1*	2.47	*V. harveyi 08-075 3T2*	2.57
		2	*V. harveyi 08-077 4T2*	2.05	*V. harveyi LMG7890*	2.37	*V. harveyi 08-077 4T2*	2.05	*V. harveyi LMG7890*	2.37
		3	*V. harveyi 04-107 1T1*	2.31	*V. harveyi 04-107 1T1*	2.47	*V. harveyi 04-107 1T1*	2.31	*V. harveyi 04-107 1T1*	2.47
V. jasicida	LMG 25398	1	*V. jasicida CECT8524*	2.37	*V. jasicida 07-078 1T2*	2.5	*V. jasicida CECT8524*	2.37	*V. jasicida 07-078 1T2*	2.5
		2	*V. jasicida CECT8524*	2.2	*V. jasicida CECT8524*	2.52	*V. harveyi VN02940* (a)	2.37	*V. jasicida CECT8524*	2.52
		3	*V. jasicida 07-078 1T2*	2.08	*V. jasicida CECT8524*	2.48	*V. jasicida 07-078 1T2*	2.08	*V. jasicida CECT8524*	2.48
	CECT8524	1	*V. jasicida CECT8524*	2.49	*V. jasicida CECT8524*	2.61	*V. jasicida CECT8524*	2.49	*V. jasicida CECT8524*	2.61
		2	*V. jasicida CECT8524*	2.44	*V. jasicida CECT8524*	2.1	*V. harveyi VN2832*(b)	2.46	*V. harveyi VN02926* (a)	2.15
		3	*V. jasicida 07-078 1T2*	2.34	*V. jasicida 07-109 T1*	2.16	*V. jasicida 07-078 1T2*	2.34	*V. harveyi VN02940* (a)	2.2
	07-040_1T2	1	*V. jasicida CECT8524*	2.33	*V. jasicida CECT8524*	2.48	*V. harveyi VN02940* (a)	2.38	*V. jasicida CECT8524*	2.48
		2	*V. jasicida CECT8524*	2.35	*V. jasicida CECT8524*	2.34	*V. harveyi VN2926*(b)	2.36	*V. harveyi VN02940* (a)	2.52
		3	*V. jasicida CECT8524*	2.4	*V. jasicida 07-109 T1*	2.44	*V. jasicida CECT8524*	2.4	*V. jasicida 07-109 T1*	2.44
	07-078_1T2	1	*V. jasicida 07-078 1T2*	2.34	*V. jasicida 07-078 1T2*	2.55	*V. harveyiVN02872*(a)	2.37	*V. jasicida 07-078 1T2*	2.55
		2	*V. jasicida 07-078 1T2*	2.26	*V. jasicida 07-109 T1*	2.46	*V. harveyiVN02872* (a)	2.43	*V. harveyiVN02872* (a)	2.6
		3	*V. jasicida CECT8524*	2.29	*V. jasicida 07-078 1T2*	2.38	*V. harveyiVN02940*(a)	2.35	*V. jasicida 07-078 1T2*	2.38
	F1A12	1	*V. jasicida CECT8524*	2.31	*V. jasicida 07-109 T1*	2.46	*V. harveyiVN02940* (a)	2.34	*V. jasicida 07-109 T1*	2.46
		2	*V. jasicida CECT8524*	2.29	*V. jasicida CECT8524*	2.38	*V. jasicida CECT8524*	2.29	*V. harveyi VN02940* (a)	2.42
		3	*V. rotiferianus 8T1 2*	1.94	*V. jasicida 07-109 T1*	2.49	*V. harveyi VN2875* (b)	1.95	*V. jasicida 07-109 T1*	2.49
	F1-C08	1	*V. jasicida 07-109 T1*	2.41	*V. jasicida 07-109 T1*	2.49	*V. jasicida 07-109 T1*	2.41	*V. jasicida 07-109 T1*	2.49
		2	*V. jasicida CECT8524*	2.24	*V. jasicida CECT8524*	2.35	*V. jasicida CECT8524*	2.24	*V. jasicida CECT8524*	2.35
		3	*V. jasicida 07-109 T1*	2.13	*V. jasicida 07-109 T1*	2.43	*V. harveyi VN02940* (a)	2.13	*V. jasicida 07-109 T1*	2.43
	F5-G08	1	V. jasicida CECT8524	2.32	*V. jasicida 07-109 T1*	2.4	*V. harveyi VN02940*(a)	2.39	*V. jasicida 07-109 T1*	2.4

		2	*V. jasicida CECT8524*	2.41	*V. jasicida CECT8524*	2.54	*V. harveyi VN02940* [a]	2.42	*V. jasicida CECT8524*	2.54
		3	*V. jasicida CECT8524*	2.44	*V. jasicida CECT8524*	2.44	*V. jasicida CECT8524*	2.44	*V. jasicida CECT8524*	2.44
	F5H11	1	*V. jasicida CECT8524*	2.39	*V. jasicida CECT8524*	2.47	*V. harveyi VN02926* [a]	2.42	*V. jasicida CECT8524*	2.47
		2	*V. jasicida CECT8524*	2.2	*V. jasicida CECT8524*	2.32	*V. harveyi VN02940* [a]	2.22	*V. harveyi VN02940*[a]	2.35
		3	*V. jasicida CECT8524*	2.27	*V. jasicida CECT8524*	2.43	*V. jasicida CECT8524*	2.27	*V. jasicida CECT8524*	2.43
V. mytili	DSM19137T	1	*V. mytili DSM19137*	1.93	*V. mytili DSM19137*	2.07	*V. mytili DSM19137*	1.93	*V. mytili DSM19137*	2.07
		2	*V. mytili DSM19137*	2.06	*V. mytili DSM19137*	2.46	*V. mytili DSM19137*	2.06	*V. mytili DSM19137*	2.46
		3	*V. mytili DSM19137*	2.49	*V. mytili DSM19137*	2.33	*V. mytili DSM19137*	2.49	*V. mytili DSM19137*	2.33
V. natriegens	CECT526T	1	*V. natriegens CECT526*	2.35	*V. natriegens CECT526*	2.43	*V. natriegens CECT526*	2.35	*V. natriegens CECT526*	2.43
		2	*V. natriegens CECT526*	1.92	*V. natriegens CECT526*	2.2	*V. natriegens VN02951* [a]	2.15	*V. natriegens VN02951* [a]	2.48
		3	*V. natriegens CECT526*	2.13	*V. natriegens CECT526*	2.51	*V. natriegens CECT526*	2.13	*V. natriegens CECT526*	2.51
V. owensii *	DSM23055Y	1	*V. owensii DSM23055*	2.38	*V. owensii DSM23055*	2.59	*V. owensii DSM23055*	2.38	*V. owensii DSM23055*	2.59
		2	*V. owensii DSM23055*	2.3	*V. owensii DSM23055* *	2.29	*V. owensii DSM23055*	2.3	*V. owensii DSM23055* *	2.29
		3	*V. owensii DSM23055*	2.43	*V. owensii DSM23055*	2.32	*V. owensii DSM23055*	2.43	*V. owensii DSM23055*	2.32
	F1-DO4	1	*V. owensii DSM23055* *	2.17	*V. owensii DSM23055* *	2.4	*V. harveyi VN3801*[b]	2.28	*V. harveyi VN3801*[b]	2.46
		2	*V. jasicida 07-109 T1* *	2.06	*V. campbellii CIP70.67*	2.00	*V. harveyi VN2926*[b]	2.25	*V. harveyi VN2822*[b]	2.15
		3	*V. rotiferianus 07-118 T1*	1.83	*V. owensii DSM23055* *	2.12	*V. rotiferianus 07-118 T1*	1.83	*V. harveyi VN3801*[b]	2.16
	OA03	1	*V. owensii DSM23055* *	2.16	*V. owensii DSM23055* *	2.43	*V. harveyi VN3801* [b]	2.42	*V. harveyi VN3801*[b]	2.6
		2	*V. jasicida CECT8524*	2.07	*V. jasicida 07-109 T1*	2.15	*V. harveyi VN02872* [a]	2.26	*V. harveyi VN3801*[b]	2.33
		3	*V. rotiferianus 7G5 1*	1.87	*V. jasicida CECT8524* *	2.18	*V. harveyi VN3801*[b]	1.9	*V. harveyi VN3801*[b]	2.35
	ZG08	1	*V. owensii DSM23055* *	2.12	*V. jasicida CECT8524* *	2.27	*V. harveyi VN3801*[b]	2.39	*V. harveyi VN3801*[b]	2.43
		2	*V. campbellii CIP70.67*	2.03	*V. jasicida 07-078 1T2*	2.09	*V. harveyi VN3801*[b]	2.15	*V. harveyi VN02940* [a]	2.31
		3	*V. owensii DSM23055*	2.02	*V. owensii DSM23055* *	2.28	*V. owensii DSM23055*	2.02	*V. harveyi VN3801*[b]	2.36
V. rotiferianus	DSM17186 T	1	*V. rotiferianus DSM17186*	2.52	*V. rotiferianus 07-103 1T3*	2.33	*V. rotiferianus DSM17186*	2.52	*V. rotiferianus 07-103 1T3*	2.33
		2	*V. rotiferianus 8T5 6*	2.27	*V. rotiferianus DSM17186*	2.59	*V. rotiferianus 8T5 6*	2.27	*V. rotiferianus DSM17186*	2.59
		3	*V. rotiferianus DSM17186*	2.07	*V. rotiferianus 07-118 T1*	2.63	*V. rotiferianus DSM17186*	2.07	*V. rotiferianus 07-118 T1*	2.63
	16-148_1T3	1	*V. rotiferianus 16-148 1T3*	2.52	*V. rotiferianus DSM17186*	2.59	*V. rotiferianus 16-148 1T3*	2.52	*V. rotiferianus DSM17186*	2.59
		2	*V. rotiferianus 16-148 1T3*	2.44	*V. rotiferianus 07-118 T1*	2.51	*V. rotiferianus 16-148 1T3*	2.44	*V. rotiferianus 07-118 T1*	2.51
		3	*V. rotiferianus 16-148 1T3*	2.25	*V. rotiferianus 16-148 1T3*	2.63	*V. rotiferianus 16-148 1T3*	2.25	*V. rotiferianus 16-148 1T3*	2.63
	7H5_2	1	*V. rotiferianus 7G5 1*	2.52	*V. rotiferianus DSM17186*	2.64	*V. rotiferianus 7G5 1*	2.52	*V. rotiferianus DSM17186*	2.64
		2	*V. rotiferianus 16-148 1T3*	2.39	*V. rotiferianus 7G5 1*	2.51	*V. rotiferianus 16-148 1T3*	2.39	*V. rotiferianus 7G5 1*	2.51
		3	*V. rotiferianus 16-148 1T3*	2.44	*V. rotiferianus 07-103 1T3*	2.51	*V. rotiferianus 16-148 1T3*	2.44	*V. rotiferianus 07-103 1T3*	2.51
	8T1_2	1	*V. rotiferianus 07-118 T1*	2.49	*V. rotiferianus 07-118 T1*	2.64	*V. rotiferianus 07-118 T1*	2.49	*V. rotiferianus 07-118 T1*	2.64
		2	*V. rotiferianus 16-148 1T3*	2.00	*V. rotiferianus DSM17186*	2.66	*V. rotiferianus 16-148 1T3*	2.00	*V. rotiferianus DSM17186*	2.66
		3	*V. rotiferianus 7H5 2*	2.45	*V. rotiferianus 16-148 1T3*	2.38	*V. rotiferianus 7H5 2*	2.45	*V. rotiferianus 16-148 1T3*	2.38
	OB06	1	*V. rotiferianus 07-118 T1*	2.54	*V. rotiferianus DSM17186*	2.44	*V. rotiferianus 07-118 T1*	2.54	*V. rotiferianus DSM17186*	2.44
		2	*V. rotiferianus 16-148 1T3*	2.37	*V. rotiferianus DSM17186*	2.43	*V. rotiferianus 16-148 1T3*	2.37	*V. rotiferianus DSM17186*	2.43

		3	*V. rotiferianus 07-118 T1*	2.34	*V. rotiferianus 07-118 T1*	2.59	*V. rotiferianus 07-118 T1*	2.34	*V. rotiferianus 07-118 T1*	2.59
	OE12	1	*V. rotiferianus 7G5 1*	2.4	*V. rotiferianus 7G5 1*	2.55	*V. rotiferianus 7G5 1*	2.4	*V. rotiferianus 7G5 1*	2.55
		2	*V. rotiferianus 16-148 1T3*	2.35	*V. rotiferianus 7G5 1*	2.49	*V. rotiferianus 16-148 1T3*	2.35	*V. rotiferianus 7G5 1*	2.49
		3	*V. rotiferianus DSM17186*	2.26	*V. rotiferianus 8T5 6*	2.52	*V. rotiferianus DSM17186*	2.26	*V. rotiferianus 8T5 6*	2.52
	OG05	1	*V. rotiferianus DSM17186*	2.55	*V. rotiferianus 7G5 1*	2.66	*V. rotiferianus DSM17186*	2.55	*V. rotiferianus 7G5 1*	2.66
		2	*V. rotiferianus 16-148 1T3*	2.46	*V. rotiferianus 16-148 1T3*	2.35	*V. rotiferianus 16-148 1T3*	2.46	*V. rotiferianus 16-148 1T3*	2.35
		3	*V. rotiferianus 07-118 T1*	2.31	*V. rotiferianus 8T6 4*	2.53	*V. rotiferianus 07-118 T1*	2.31	*V. rotiferianus 8T6 4*	2.53
	OG10	1	*V. rotiferianus 16-148 1T3*	2.41	*V. rotiferianus DSM17186*	2.54	*V. rotiferianus 16-148 1T3*	2.41	*V. rotiferianus DSM17186*	2.54
		2	*V. rotiferianus 16-148 1T3*	2.41	*V. rotiferianus 16-148 1T3*	2.47	*V. rotiferianus 16-148 1T3*	2.41	*V. rotiferianus 16-148 1T3*	2.47
		3	*V. rotiferianus 7H5 2*	2.34	*V. rotiferianus 7H5 2*	2.5	*V. rotiferianus 7H5 2*	2.34	*V. rotiferianus 7H5 2*	2.5
	OHD8	1	*V. rotiferianus 8T5 6*	2.55	*V. rotiferianus DSM17186*	2.62	*V. rotiferianus 8T5 6*	2.55	*V. rotiferianus DSM17186*	2.62
		2	*V. rotiferianus 16-148 1T3*	2.29	*V. rotiferianus 8T1 2*	2.55	*V. rotiferianus 16-148 1T3*	2.29	*V. rotiferianus 8T1 2*	2.55
		3	*V. rotiferianus 16-148 1T3*	2.32	*V. rotiferianus 16-148 1T3*	2.47	*V. rotiferianus 16-148 1T3*	2.32	*V. rotiferianus 16-148 1T3*	2.47
V. sagamiensis	DSM25651	1	*V. sagamiensis DSM25651*	2.4	*V. sagamiensis DSM25651*	2.51	*V. sagamiensis DSM25651*	2.4	*V. sagamiensis DSM25651*	2.51
		2	*V. sagamiensis DSM25651*	2.19	*V. sagamiensis DSM25651*	2.3	*V. sagamiensis DSM25651*	2.19	*V. sagamiensis DSM25651*	2.3
		3	*V. sagamiensis DSM25651*	1.78	*V. sagamiensis DSM25651*	2.09	*V. sagamiensis DSM25651*	1.78	*V. sagamiensis DSM25651*	2.09
V. mediterranei	DSM13773	1	*V. mediterranei DSM13773T*	2.44	*V. mediterranei DSM13773T*	2.43	*V. mediterranei DSM13773T*	2.44	*V. mediterranei DSM13773T*	2.43
		2	*V. mediterranei DSM13773T*	2.23	*V. mediterranei DSM13773T*	2.18	*V. mediterranei DSM13773T*	2.23	*V. mediterranei DSM13773T*	2.18
		3	*V. mediterranei DSM13773T*	2.31	*V. mediterranei DSM13773T*	2.41	*V. mediterranei DSM13773T*	2.31	*V. mediterranei DSM13773T*	2.41
V. bivalvicida *	CECT8855T	1	*V. bivalvicida CECT8855*	2.3	*V. bivalvicida CECT8855*	2.34	*V. bivalvicida CECT8855*	2.3	*V. bivalvicida CECT8855*	2.34
		2	*V. bivalvicida 14-106 49T1* *	2.38	*V. bivalvicida 14-106 49T1* *	2.42	*V. bivalvicida 14-106 49T1* *	2.38	*V. bivalvicida 14-106 49T1* *	2.42
		3	*V. bivalvicida CECT8856* *	2.18	*V. bivalvicida CECT8856* *	2.2	*V. bivalvicida CECT8856* *	2.18	*V. bivalvicida CECT8856* *	2.2
	14-105 3T2	1	*V. bivalvicida 14-106 49T1*	2.46	*V. bivalvicida 14-106 1T1*	2.43	*V. bivalvicida 14-106 49T1*	2.46	*V. bivalvicida 14-106 1T1*	2.43
		2	*V. bivalvicida 14-106 49T1* *	2.46	*V. bivalvicida 14-106 49T1* *	2.53	*V. bivalvicida 14-106 49T1* *	2.46	*V. bivalvicida 14-106 49T1* *	2.53
		3	*V. bivalvicida CECT8856*	2.3	*V. bivalvicida CECT8856* *	2.21	*V. bivalvicida CECT8856*	2.3	*V. bivalvicida CECT8856* *	2.21
	14-106_1T1	1	*V. bivalvicida 14-106 49T1*	2.42	*V. bivalvicida 14-106 49T1* *	2.5	*V. bivalvicida 14-106 49T1*	2.42	*V. bivalvicida 14-106 49T1* *	2.5
		2	*V. bivalvicida CECT8856*	2.37	*V. bivalvicida 14-105 2T1* *	2.49	*V. bivalvicida CECT8856*	2.37	*V. bivalvicida 14-105 2T1* *	2.49
		3	*V. bivalvicida CECT8856* *	2.02	*V. bivalvicida 14-105 3T1* *	2.55	*V. bivalvicida CECT8856* *	2.02	*V. bivalvicida 14-105 3T1* *	2.55
	14-106_49T2	1	*V. bivalvicida 14-106 49T1*	2.4	*V. bivalvicida 14-106 49T1*	2.36	*V. bivalvicida 14-106 49T1*	2.4	*V. bivalvicida 14-106 49T1*	2.36
		2	*V. bivalvicida 14-106 49T1* *	2.36	*V. bivalvicida 14-105 2T1*	2.48	*V. bivalvicida 14-106 49T1* *	2.36	*V. bivalvicida 14-105 2T1*	2.48
		3	*V. bivalvicida CECT8856* *	2.13	*V. bivalvicida 14-106 49T1*	2.57	*V. bivalvicida CECT8856* *	2.13	*V. bivalvicida 14-106 49T1*	2.57
V. brasiliensis	DSM17184T	1	*V. brasiliensis DSM17184T*	2.51	*V. brasiliensis DSM17184T*	2.6	*V. brasiliensis DSM17184T*	2.51	*V. brasiliensis DSM17184T*	2.6
		2	*V. brasiliensis DSM17184T*	2.34	*V. brasiliensis DSM17184T*	2.33	*V. brasiliensis DSM17184T*	2.34	*V. brasiliensis DSM17184T*	2.33
		3	*V. brasiliensis DSM17184T*	2.03	*V. brasiliensis DSM17184T*	2.54	*V. brasiliensis DSM17184T*	2.03	*V. brasiliensis DSM17184T*	2.54
V. europaeus *	CECT8136	1	*V. europaeus 07-121 1T1*	2.54	*V. europaeus 07-108 T1*	2.4	*V. europaeus 07-121 1T1*	2.54	*V. europaeus 07-108 T1*	2.4
		2	*V. europaeus 07-108 T1* *	2.25	*V. europaeus CECT8136* *	2.42	*V. europaeus 07-108 T1* *	2.25	*V. europaeus CECT8136* *	2.42
		3	*V. europaeus 07-108 T1*	2.18	*V. europaeus CECT8136*	2.09	*V. europaeus 07-108 T1*	2.18	*V. europaeus CECT8136*	2.09
	04-002_1T2	1	*V. europaeus 07-108 T1*	2.51	*V. europaeus 07-119 T1* *	2.42	*V. europaeus 07-108 T1*	2.51	*V. europaeus 07-119 T1* *	2.42

		2	V. europaeus 07-117 T1	*	2.46	V. europaeus 07-108 T1	*	2.62	V. europaeus 07-117 T1		2.46	V. europaeus 07-108 T1	*	2.62
		3	V. europaeus 07-121 1T1		2.39	V. europaeus 07-119 T1		2.55	V. europaeus 07-121 1T1		2.39	V. europaeus 07-119 T1		2.55
	14-106 32T3	1	V. tubiashii DSM19142	*	2.53	V. europaeus 07-038 2T2	*	2.27	V. tubiashii DSM19142	*	2.53	V. europaeus 07-038 2T2	*	2.27
		2	V. tubiashii DSM19142	*	2.31	V. tubiashii DSM19142	*	2.31	V. tubiashii DSM19142	*	2.31	V. tubiashii DSM19142	*	2.31
		3	V. tubiashii DSM19142	*	2.48	V. europaeus 07-121 1T1	*	2.04	V. tubiashii DSM19142	*	2.48	V. europaeus 07-121 1T1	*	2.04
	14-106 37T1	1	V. tubiashii DSM19142	*	2.43	V. tubiashii DSM19142	*	2.27	V. tubiashii DSM19142	*	2.43	V. tubiashii DSM19142	*	2.27
		2	V. tubiashii DSM19142	*	2.31	V. tubiashii DSM19142	*	2.46	V. tubiashii DSM19142	*	2.31	V. tubiashii DSM19142	*	2.46
		3	V. tubiashii DSM19142		1.94	V. tubiashii DSM19142	*	2.21	V. tubiashii DSM19142		1.94	V. tubiashii DSM19142	*	2.21
	14-107 4T1	1	V. tubiashii DSM19142	*	2.5	V. europaeus 07-110 T1	*	2.17	V. tubiashii DSM19142	*	2.5	V. europaeus 07-110 T1	*	2.17
		2	V. tubiashii DSM19142	*	2.38	V. tubiashii DSM19142	*	2.47	V. tubiashii DSM19142	*	2.38	V. tubiashii DSM19142	*	2.47
		3	V. europaeus 07-108 T1		1.88	V. tubiashii DSM19142	*	2.14	V. europaeus 07-108 T1		1.88	V. tubiashii DSM19142	*	2.14
	14-107 7T2	1	V. tubiashii DSM19142	*	2.51	V. europaeus 07-110 T1	*	2.26	V. tubiashii DSM19142	*	2.51	V. europaeus 07-110 T1	*	2.26
		2	V. tubiashii DSM19142	*	2.37	V. tubiashii DSM19142	*	2.38	V. tubiashii DSM19142	*	2.37	V. tubiashii DSM19142	*	2.38
		3	V. tubiashii DSM19142	*	2.52	V. europaeus 07-121 1T1		2.46	V. tubiashii DSM19142	*	2.52	V. europaeus 07-121 1T1		2.46
	16-148 1T1	1	V. europaeus 07-108 T1		2.53	V. europaeus 07-038 2T2		2.28	V. europaeus 07-108 T1		2.53	V. europaeus 07-038 2T2		2.28
		2	V. europaeus 07-110 T1	*	2.48	V. europaeus 07-116 T1	*	2.4	V. europaeus 07-110 T1	*	2.48	V. europaeus 07-116 T1	*	2.4
		3	V. europaeus 07-108 T1		2.51	V. europaeus 07-121 1T1		2.54	V. europaeus 07-108 T1		2.51	V. europaeus 07-121 1T1		2.54
V. hepatarius *	DSM19134	1	V. hepatarius DSM19134	*	2.36	V. orientalis 8H6 11	*	2.3	V. hepatarius DSM19134	*	2.36	V. orientalis 8H6 11	*	2.3
		2	V. orientalis LMG7897T	*	2.57	V. orientalis LMG7897T	*	2.54	V. orientalis LMG7897T	*	2.57	V. orientalis LMG7897T	*	2.54
		3	V. orientalis 8Z-12	*	2.02	V. hepatarius DSM19134	*	2.49	V. orientalis 8Z-12	*	2.02	V. hepatarius DSM19134	*	2.49
V. orientalis	LMG7897T	1	V. orientalis LMG7897T		2.54	V. orientalis LMG7897T		2.35	V. orientalis LMG7897T		2.54	V. orientalis LMG7897T		2.35
		2	V. orientalis LMG7897T		2.42	V. orientalis LMG7897T		2.46	V. orientalis LMG7897T		2.42	V. orientalis LMG7897T		2.46
		3	V. orientalis LMG7897T		2.07	V. orientalis LMG7897T		2.04	V. orientalis LMG7897T		2.07	V. orientalis LMG7897T		2.04
V. sinaloensis	CECT7298T	1	V. sinaloensis CECT7298		2.36	V. sinaloensis CECT7298		2.07	V. sinaloensis CECT7298		2.36	V. sinaloensis CECT7298		2.07
		2	V. sinaloensis CECT7298		2.28	V. sinaloensis CECT7298		2.45	V. sinaloensis CECT7298		2.28	V. sinaloensis CECT7298		2.45
		3	V. sinaloensis CECT7298		2.02	V. sinaloensis CECT7298		2.21	V. sinaloensis CECT7298		2.02	V. sinaloensis CECT7298		2.21
V. tubiashii *	DSM19142T	1	V. tubiashii DSM19142	*	2.4	V. tubiashii DSM19142	*	2.45	V. tubiashii DSM19142	*	2.4	V. tubiashii DSM19142	*	2.45
		2	V. tubiashii DSM19142		2.58	V. tubiashii DSM19142		2.71	V. tubiashii DSM19142		2.58	V. tubiashii DSM19142		2.71
		3	V. tubiashii DSM19142		2.57	V. tubiashii DSM19142	*	2.21	V. tubiashii DSM19142		2.57	V. tubiashii DSM19142	*	2.21
	14-106 35T1	1	V. tubiashii DSM19142T/ V. europaeus 07-121 1T1	*	2.4	V. tubiashii DSM19142T		2.37	V. tubiashii DSM19142T/ V. europaeus 07-121 1T1	*	2.4	V. tubiashii DSM19142T		2.37
		2	V. tubiashii DSM19142		2.47	V. tubiashii DSM19142	*	2.43	V. tubiashii DSM19142		2.47	V. tubiashii DSM19142	*	2.43
		3	V. europaeus 07-110 T1		2.02	V. tubiashii DSM19142		1.96	V. europaeus 07-110 T1		2.02	V. tubiashii DSM19142		1.96
	14-107 6T2	1	V. tubiashii DSM19142	*	2.42	V. tubiashii DSM19142	*	2.2	V. tubiashii DSM19142	*	2.42	V. tubiashii DSM19142	*	2.2
		2	V. europaeus CECT8136	*	2.17	V. tubiashii DSM19142		2.33	V. europaeus CECT8136	*	2.17	V. tubiashii DSM19142		2.33
		3	V. europaeus 07-108 T1		2.04	V. tubiashii DSM19142		2.39	V. europaeus 07-108 T1		2.04	V. tubiashii DSM19142		2.39
	LMG11229	1	V. tubiashii DSM19142	*	2.56	V. tubiashii DSM19142		2.49	V. tubiashii DSM19142	*	2.56	V. tubiashii DSM19142		2.49
		2	V. tubiashii DSM19142	*	2.2	V. tubiashii DSM19142		2.51	V. tubiashii DSM19142	*	2.2	V. tubiashii DSM19142		2.51

		3	*V. tubiashii DSM19142* *	2.14	*V. tubiashii DSM19142* *	2.45	*V. tubiashii DSM19142* *	2.14	*V. tubiashii DSM19142* *	2.45
V. cortegadensis	DSM102805	1	*V. cortegadensis DSM102805*	2.24	*V. cortegadensis DSM102805*	2.52	*V. cortegadensis DSM102805*	2.24	*V. cortegadensis DSM102805*	2.52
		2	*V. cortegadensis DSM102805*	2.47	*V. cortegadensis DSM102805*	2.27	*V. cortegadensis DSM102805*	2.47	*V. cortegadensis DSM102805*	2.27
		3	*V. cortegadensis DSM102805*	2.17	*V. cortegadensis DSM102805*	2.05	*V. cortegadensis DSM102805*	2.17	*V. cortegadensis DSM102805*	2.05
V. tapetis tapetis	RD0202	1	*V. tapetis tapetis LP2*	2.49	*V. tapetis tapetis LP2*	2.53	*V. tapetis tapetis LP2*	2.49	*V. tapetis tapetis LP2*	2.53
		2	*V. tapetis tapetis LP2*	2.46	*V. tapetis tapetis IS5*	1.73	*V. tapetis tapetis LP2*	2.46	*V. tapetis tapetis IS5*	1.73
		3	*V. tapetis tapetis LP2*	2.05	*V. tapetis tapetis LP2*	2.22	*V. tapetis tapetis LP2*	2.05	*V. tapetis tapetis LP2*	2.22
	RD0705	1	*V. tapetis tapetis IS9*	2.43	*V. tapetis tapetis 2.3*	2.57	*V. tapetis tapetis IS9*	2.43	*V. tapetis tapetis 2.3*	2.57
		2	*V. tapetis tapetis IS5*	2.44	*V. tapetis tapetis IS5*	2.28	*V. tapetis tapetis IS5*	2.44	*V. tapetis tapetis IS5*	2.28
		3	*V. tapetis tapetis IS5*	2.17	*V. tapetis tapetis RP 1703*	2.36	*V. tapetis tapetis IS5*	2.17	*V. tapetis tapetis RP 1703*	2.36

B.

			~~VibrioBase LNR~~EnviBase				~~VibrioBase LNR~~ EnviBase+ Bruker ~~Biotyper~~ + Erler databases			
**Specie**	**Strain**	**Lab**	**Direct transfer**	**Best Score**	**Protein extraction**	**Best Score**	**Direct transfer**	**Best Score**	**Protein extraction**	**Best Score**
Alteromonas mackleodi	LMG20615	1	*V. aestuarianus 09-045 5T1*	1.13	*V. aestuarianus 11-183 10T2*	0.99	*Flavobacterium hydatis DSM2063T*	1.28	*Campylobacter jejuni MB6111*[(a)]	1.25
		2	*V. aestuarianus 03-146 1B1*	0.73	*V. aestuarianus 12-146 3T1*	0.94	*Malassezia pachydermatis CBS1879T*[(a)]	1.19	*Pseudomonas poae DSM14936T*[(a)]	1.24
		3	*V. aestuarianus 10-075 3T2*	0.95	*V. aestuarianus edule 12-122 2T3*	1.02	*Bacillus farraginis DSM16013T*[(a)]	1.21	*Rhizobium radiobacter B165*[(a)]	1.36
V. anguillarum	LMG4437T	1	*V. aestuarianus 09-045 5T1*	1.96	*V. aestuarianus 07-139 1T1*	2.03	*V. anguillarum DSM11323*	2.23	*V. anguillarum DSM21597T*	2.38
		2	*V. aestuarianus 12-122 2T3*	1.85	*V. aestuarianus 09-045 5T1*	1.96	*V. anguillarum DSM21597T*	2.37	*V. anguillarum DSM21597T*	2.47
		3	*V. aestuarianus 11-081 4MT2*	1.88	*V. aestuarianus 08-081 5T1*	1.96	*Listonella anguillarum VN2855*	1.9	*V. anguillarum DSM21597T*	2.22
V. azureus	LMG25266	1	*V. jasicida CECT8524*	1.98	*V. jasicida 07-109 T1*	1.93	*V. harveyi VN2829*[(b)]	1.99	*V. jasicida 07-109 T1*	1.93
		2	*V. rotiferianus 7G5 1*	1.82	*V. jasicida 07-081 2T2*	1.89	*V. harveyi VN2832*[(b)]	1.85	*V. harveyi VN02926*[(a)]	1.95
		3	*V. harveyi LMG7890*	1.57	*V. jasicida 07-109 T1*	1.71	*V. harveyi VN2832*[(b)]	1.79	*V. harveyi VN2872*[(b)]	1.84
V. barjei	LMG29358	1	*V. mediterranei DSM13773T*	2.29	*V. mediterranei DSM13773*	2.34	*V. mediterranei DSM13773*	2.29	*V. mediterranei DSM13773*	2.34
		2	*V. mediterranei DSM13773*	2.1	*V. mediterranei DSM13773*	2.24	*V. mediterranei DSM13773*	2.1	*V. mediterranei DSM13773*	2.24
		3	*V. mediterranei DSM13773*	1.95	*V.mediterranei DSM13773*	2.33	*V. mediterranei DSM13773*	1.95	*V. mediterranei DSM13773*	2.33
V. chagasii	OF05	1	*V. sagamiensis DSM25651*	1.6	*V. aestuarianus 16-055 5T1*	1.68	*V. gigantis VN3815*[(b)]	2.34	*V. gigantis VN3815*[(b)]	2.24
		2	*V. aestuarianus 12-147 1T1*	1.22	*V. aestuarianus 09-045 5T1*	1.6	*V. gigantis VN3815*[(b)]	1.95	*V. gigantis VN3815*[(b)]	2.26
		3	*V. rotiferianus 07-115 T1*	1.45	*V. aestuarianus 11-081 4MT2*	1.54	*V. gigantis VN3815*[(b)]	2.13	*V. gigantis VN3815*[(b)]	2.34

V. diabolicus	14-079_2T1	1	*V. alginolyticus* LMG4409T	2.3	*V. alginolyticus* LMG4409T	2.39	*V. alginolyticus* VN2565[b]	2.44	*V. alginolyticus* VN3355(b)	2.47
		2	*V. alginolyticus* LMG4409T	2.12	*V. alginolyticus* LMG4409T	2.16	*V. alginolyticus* VN2836[b]	2.36	*V. alginolyticus VN2559(b)*	2.28
		3	*V. rotiferianus* DSM17186	1.49	*V. alginolyticus* LMG4409	2.27	*V. harveyi* VN2921	1.61	*V. alginolyticus* VN2836(b)	2.37
V. galatheae	LMG28895 T	1	*V. tubiashii* DSM19142T	2.00	*V. europaeus* 07-120 T1	1.75	*V. tubiashii* DSM19142 *T*	2	*V. europaeus* 07-120 T1	1.75
		2	*V. bivalvicida* CECT8856	1.93	*V. europaeus* 07-108 T1	2.04	*V. bivalvicida* CECT8856	1.93	*V. europaeus* 07-108 T1	2.04
		3	*V. brasiliensis* DSM *17184*	1.52	*V. tubiashii* DSM19142T	2.02	*V. brasiliensis* DSM 17184	1.52	*V. tubiashii* DSM19142 T	2.02
V. hyuagaensis	LMG28466	1	*V. jasicida* 07-078 1T2	2.2	*V. jasicida* CECT8524	2.46	*V. harveyi* VN02940 [a]	2.29	*V. jasicida* CECT8524	2.46
		2	*V. jasicida* CECT8524	1.84	*V. jasicida* CECT8524	2.46	*V. harveyi* VN2818 [b]	2	*V. harveyi* VN02940 (a)	2.57
		3	*V. jasicida* CECT8524	1.92	*V. jasicida* 07-078 1T2	2.51	*V. jasicida* CECT8524	1.92	*V. jasicida* 07-078 1T2	2.51
V. nereis	LMG3895T	1	*V. neptunius* 09-123 1T2	1.73	*V. tubiashii* DSM19142T	1.73	*V. nereis* LMG3895T	1.74	*V. nereis* LMG3895T	1.9
		2	*V. neptunius* 09-123 1T2	1.8	*V. coralliilyticus* 06-210	1.72	*V. nereis* LMG3895T	1.84	*V. nereis* LMG3895T	1.76
		3	*V. neptunius* 09-123 1T2	1.55	*V. coralliilyticus* LMG19607T	1.64	*V. nereis* LMG3895T	1.8	*V. coralliilyticus* LMG19607T	1.64
V. salilacus	JCM 19265	1	*V. ostreicida* CECT7399	1.64	*V. sinaloensis* CECT7298	1.53	*V. ostreicida* DSM21434[a]	1.64	*V. ostreicida* DSM21434[a]	1.64
		2	*V. ostreicida* CECT 7399	1.76	*V. ostreicida* CECT 7399	1.83	*V. pacinii* LMG19999T HAM[a]	1.88	*V. ostreicida* CECT 7399	1.83
		3	*V. europaeus* 07-110T1	1.57	*V. mytili* DSM19137	1.75	*V. pacini* LMG 19999T HAM	1.59	*V. mytili* DSM19137	1.75
V. splendidus	LMG4042	1	*V. tapetis tapetis* LP2	1.5	*V. aestuarianus* 12-122 3T3	1.59	*V. tasmaniensis* VN3824[b]	2.16	*V. tasmaniensis* VN3824[b]	2.2
		2	*V. aestuarianus* 12-147 1T1	1.38	*V. alginolyticus* LMG4409T	1.25	*V. tasmaniensis* VN3824[b]	2.16	*V. kanaloe* VN3817	1.77
		3	*V. aestuarianus* 15-064 4T2	1.33	*V. aestuarianus* 16-055 5T1	1.5	*V. tasmaniensis* VN3824	1.76	*V. tasmaniensis* VN3824[b]	2.03
V. xuii	DSM17185 T	1	*V. alginolyticus* LMG4409T	1.79	*V. jasicida* CECT8524	1.76	*V. xuii* VN3825[b]	2.5	*V. xuii* VN3825[b]	2.5
		2	*V. campbellii* CIP 70.67	1.69	*V. jasicida* CECT8524	1.73	*V. xuii* VN3825[b]	2.48	*V. xuii* VN3825[b]	2.22
		3	*V. alginolyticus* LMG4409T	1.82	*V. campbellii* CIP70.67	1.94	*V. xuii* VN3825[b]	2.37	*V. xuii* VN3825[b]	2.56
V. jasicida-like	F5-C01	1	*V. rotiferianus*7G5 1	2.06	*V. rotiferianus* 7G5 1	1.9	*V. rotiferianus* 7G5 1	2.06	*V. harveyi* VN3801	1.93
		2	*V. rotiferianus*DSM17186	1.95	*V. jasicida* 07-078 1T2	2.1	*V. rotiferianus* DSM17186	1.95	*V. jasicida* 07-078 1T2	2.1
		3	*V. rotiferianus*8T1 2	1.87	*V. jasicida* 07-078 1T2	2.01	*V. rotiferianus* 8T1 2	1.87	*V. harveyi* VN3801[b]	2.03

Score values: 2.300–3.000 (Highly probable species-level identification); 2.000–2.229 (secure genus-level identification and a probable species-level identification); ≥ 1.700–1.999 (probable genus-level identification) and < 1.700 (unreliable identification). ***** Strains for which it is impossible to conclude; Incorrect identifications despite scores >2 are indicated by a red text on a green background.[(a)]Classification corresponding to strains issued from Bruker database; [(b)] Classification corresponding to the strains issued from Erler's database.
